# An Improved Marine Predators Algorithm With Fuzzy Entropy for Multi-Level Thresholding: Real World Example of COVID-19 CT Image Segmentation

**DOI:** 10.1109/ACCESS.2020.3007928

**Published:** 2020-07-08

**Authors:** Mohamed Abd Elaziz, Ahmed A. Ewees, Dalia Yousri, Husein S. Naji Alwerfali, Qamar A. Awad, Songfeng Lu, Mohammed A. A. Al-Qaness

**Affiliations:** Department of MathematicsFaculty of ScienceZagazig University 68799 Zagazig 44519 Egypt; Department of ComputerDamietta University 435384 Damietta 34511 Egypt; Electrical Engineering DepartmentFaculty of EngineeringFayoum University 158401 Faiyum 63514 Egypt; School of Computer Science and TechnologyHuazhong University of Science and Technology 12443 Wuhan 430074 China; Hubei Engineering Research Center on Big Data Security, School of Cyber Science and EngineeringHuazhong university of Science and Technology 12443 Wuhan 430074 China; State Key Laboratory for Information Engineering in Surveying, Mapping, and Remote SensingWuhan University 12390 Wuhan 430079 China

**Keywords:** Image segmentation, multi-level thresholding, moth-?ame optimization (MFO), marine predators algorithm (MPA), COVID-19, swarm intelligence

## Abstract

Medical imaging techniques play a critical role in diagnosing diseases and patient healthcare. They help in treatment, diagnosis, and early detection. Image segmentation is one of the most important steps in processing medical images, and it has been widely used in many applications. Multi-level thresholding (MLT) is considered as one of the simplest and most effective image segmentation techniques. Traditional approaches apply histogram methods; however, these methods face some challenges. In recent years, swarm intelligence methods have been leveraged in MLT, which is considered an NP-hard problem. One of the main drawbacks of the SI methods is when searching for optimum solutions, and some may get stuck in local optima. This because during the run of SI methods, they create random sequences among different operators. In this study, we propose a hybrid SI based approach that combines the features of two SI methods, marine predators algorithm (MPA) and moth-?ame optimization (MFO). The proposed approach is called MPAMFO, in which, the MFO is utilized as a local search method for MPA to avoid trapping at local optima. The MPAMFO is proposed as an MLT approach for image segmentation, which showed excellent performance in all experiments. To test the performance of MPAMFO, two experiments were carried out. The first one is to segment ten natural gray-scale images. The second experiment tested the MPAMFO for a real-world application, such as CT images of COVID-19. Therefore, thirteen CT images were used to test the performance of MPAMFO. Furthermore, extensive comparisons with several SI methods have been implemented to examine the quality and the performance of the MPAMFO. Overall experimental results confirm that the MPAMFO is an efficient MLT approach that approved its superiority over other existing methods.

## I. Introduction

With the fast spread of the new coronavirus, COVID-19, researchers are trying to address different aspects related to this new virus. One of the most important issues is diagnosing COVID-19 using different tests, including the real-time polymerase chain reaction (RTPCR), and chest CT. The RT-PCR is a time-consuming test, and also it faces false-negative diagnosing [1]. Therefore, chest CT scans may play an important role in diagnosing COVID-19. Medical imaging technologies have been implemented in different diseases diagnosing. Image segmentation is an essential technique in image processing, and it is an important procedure in various image and vision applications, which can efficiently detect a region of interest (ROI) form other outsides. It is applied to classify image pixels into different classes which contain similar properties, such as brightness, gray level, contrast, texture, and color. Also, it is able to extract important features, such as texture and shape of tissues [2]

The segmentation process has been applied in various fields and applications, for instance, medical image [3], remote sensing [4], video surveillance [5] and other applications [6], [7]. Several types of image segmentation techniques have been proposed and applied, such as clustering [8], thresholding [9], edge detection [10], and edge detection [10].

Thresholding is considered one of the most important image segmentation techniques, which is implemented to segment images depended on the information in the global gray values of the image histogram [11]. In general, there are two types of thresholding, called bi-level thresholding (BLT) and multi-level thresholding (MLT). For BLT, an image is divided into two classes, in which one class contains pixels with gray levels above a threshold, and the other class contains the rest [11]. However, the BLT faces a challenge in case of a given image has more than two classes. Therefore, the MLT can solve this challenge by implementing the subdivision of a given image into more classes.

Traditional MLT segmentation methods are based on the image grey-level histogram [12] by minimizing or maximizing the fitness functions, for example, entropy [13] and Otsu [14]. However, there are certain limitations and shortcomings in the performance of traditional MLT techniques. For example, they are time-consuming, especially when the number of threshold levels is increased. In addition, they easily stuck at a local point. Therefore, optimization methods have been widely employed to enhance MLT since MLT can be considered as NP-hard problem. In the recent decade, several optimization methods have been used to improve MLT, such as MFO [15], cuckoo search (CS) [16], [17], ant colony optimizer (ACO) [18], chaotic bat algorithm (CBA) [19], WOA [20], and firefly algorithm (FA) [21]–[24].

Although the optimization algorithms mentioned above showed good performances in MLT since they can find the optimal threshold value, they face some challenges, such as getting stuck at local optima or suffer from slow convergence [25]–[30]. In general, according to the NFL (No free lunch) theorems, no optimization method can be the best for solving all problems. In general, some optimization methods have good exploitation ability, and some have good exploration ability [31]. To address these issues, various hybrid optimization methods have been proposed. For example, a hybrid of FA and social spider optimization (SSO) was proposed by [32] for MLT image segmentation. The new hybrid optimization method achieved better results than individual optimization methods. In [33], an MLT image segmentation method based on a hybrid of PSO and BFO is proposed. Eight images were used to test the hybrid model and reached good results for both MLT and BLT. More so, MLT and optimization methods have been applied for different medical image segmentation, such as CT images [34]–[36], MR images [37], [38], MRI image [20], [39].

Following the hybridization concepts, in this study, we propose an efficient MLT method based on an improved marine predators algorithm (MPA) for image segmentation. The MFO is employed as a local search for the MPA to improve its performance. The proposed method, MPAMFO, is an efficient hybrid optimization method for MLT that overcomes the shortcomings of individual optimization methods using the power of both MPA and MFO. The MPA is a new nature-inspired optimization algorithm proposed by Faramarzi *et al.* [40]. It is inspired by the movements of Lévy and Brownian in ocean predators. Twenty-nine engineering problems were used to test its performance, and it showed high performances in various optimization problems. MPA has some merits, such as its requirement for the least number of tunable parameters, its simplicity in the implementation, and flexibility in modifying the basic MPA version that attracted Yousri *et al.* [41] to apply basic MPA for photovoltaic reconfiguration. Whereas, the shortage of the MPA while the exploration stage for the search space motivated Abdel-Basset *et al.* [42] to modify the MPA by using ranking-based diversity reduction (RDR) methodology to discover better solutions while applied with for COVID-19 Detection Model. Accordingly, proposing a robust MPA variant is a challenged door to tackle its shortage.

The MFO is a nature-inspired optimization method proposed by [43], which simulates the behaviors of the moth for path navigation. In recent years, it has been applied to solve various optimization problems. Kotary and Nanda [44] applied MFO to improve distributed data clustering in wireless sensor networks (WSN). The main function of the diffusion MFO is by minimizing intracluster distance, which results in determining the optimal partition of each sensor node. Ewees *et al.* [45] used the MFO to improve Arabic handwritten letters recognition. They applied the MFO as a feature selector, which achieved a high accuracy rate compared to previous approaches. In [46], MFO was applied to enhance ANFIS model to forecast the number of confirmed cases of the new coronavirus (COVID-19). In [47], a feature selection mechanism based on differential evolution and MFO is proposed. They tested the proposed hybrid model with different CEC2005 benchmark problems, and they found that the proposed method outperformed several existing methods. Zhao *et al.* [48] applied MFO to optimize the grey model (1,1) with a rolling mechanism for forecasting electricity consumption in Inner Mongolia. The evaluation results showed that MFO improved forecasting performance. It has also been applied for solving different mathematical problems, for example, multi-objective problems [49], binary problems [50], and and other applications [51], [52]. By inspecting the literature, one can observe that implementing the logarithmic spiral function in MFO in the phase of the moths update their position concerning the flame strengthened the searching ability of the algorithm. Moreover, MFO simplicity and flexibility motivated numerous researchers have been working on it.

Motivated by the merits of the MFO of its ability to discover the search space efficiently and demerit of MPA in detecting better solutions in the exploration phase, in this work, a new hybrid version of MPA is based on MFO has been introduced. The main idea of the proposed hybrid MPA version by MFO (MPAMFO) is to enhance the exploration ability of the MPA using the operators of the MFO algorithm. This achieved by making the agents/solutions be competitive in the exploration phase by using the probability of the fitness value of each solution to determine either the operators of MPA or MFO will be used to update the value of the current agent, while the exploitation phase is performed similarly to the traditional MPA.

In this paper, we evaluate the MPAMFO using two experiments series. In the first experiment series, we used a group of ten images. These images were widely used in previous studies to test various segmentation methods. Moreover, to implement MPAMFO in a real-world application, we test it to segment chest CT images of COVID-19 [53]. The performance of both experiment series showed that the MPAMFO is an efficient segmentation method that can be applied in various segmentation applications including medical images.

The main contributions of this study can be summarized as:

1) We propose an MLT method for image segmentation based on a modified version of the new optimization method, called MPA.
2) The MFO operators are employed to improve the exploitation ability of the MPA.
3) We test the performance of the proposed method in two experiment series, using ten gray-scale popular images and thirteen CT images of COVID-19. Moreover, we compared it to several state-of-art methods.

The rest of this paper is organized as follows. Section II presents some of the existing works of the MLT and optimization methods in image segmentation, including medical images. In Section III, we present the problem definition and the preliminaries of MPA and MFO. The proposed method is described in Section V. The experimental evaluation and comparisons are presented in Section VI. In Section VII, we conclude the paper.

## II. Related Work

Mousavirad and Ebrahimpour-Komleh [54] proposed an MLT approach using Human Mental Search (HMS). They applied Kapur and Otsu as objective functions. The HMS was compared to several optimization methods, and it showed significant performance. In [55], several MH algorithms are used for MLT, such as WOA, GWO, CS, biogeography-based optimization, cuckoo optimization algorithm, teaching–learning-based optimization, imperialist competitive algorithm, and gravitational search algorithm. In the same context, the authors in [56] applied different optimization algorithms for MLT. Monisha *et al.* [57] employed Social Group Optimization for MLT for RGB images. Also, Bhandari [58] presented a new beta differential evolution (BDE) for color image MLT.

Huang and Wang [59] proposed an MLT method based on the quantum particle swarms algorithm (QPSO) algorithm for image segmentation. They used Otsu’s fitness function. They concluded that compared to traditional methods, the QPSO improved both accuracy and speed. Qin *et al.* [60] employed the subspace elimination optimization (SSEO) for MLT image segmentation. They applied the SSEO for four different images, and they compared it to the particle swarm optimization (PSO). They found that SSEO has better performance in all tested images. Both moth-flame optimization (MFO) algorithm and whale optimization algorithm (WOA) were used for MLT in [61]. The authors used Otsu’s was used as the fitness function, and they test both WOA and MFO using several images. They concluded that MFO had better performance than WOA. Farshi [62] proposed an MLT method based on animal migration optimization (AMO) algorithm. Different images were used to test the performance of the AMO algorithm, and it was compared to several optimization methods, such as PSO, bacterial foraging algorithm (BFA), and genetic algorithm (GA). As the author mentioned, the AMO algorithm provided better results. In [63], an MLT method based on electromagnetism- like mechanism optimization (EMO) and Renyi’s entropy is proposed for image segmentation. The evaluation results showed that EMO could find the optimal threshold value better than several existing optimization methods.

Tuba *et al.* [64] proposed an MLT method based on the fireworks algorithm for image segmentation. They evaluated the proposed method using several images, and it showed good performance in all tested images. In [9], an MLT method based on PSO and maximum entropy is proposed. The PSO showed good performances in several tested images compared to traditional methods. Ali *et al.* [65] proposed an improved differential evolution (DE) called synergetic DE (SDE) for MLT image segmentation. Their evaluation outcomes showed that the SED could perform better than other MLT methods in terms of reaching the optimal threshold value. The galaxy-based search algorithm (GbSA) was applied by [66] for MLT maximizing Otsu’s fitness function, and it approved its good performance to determine the optimal thresholding value. Ewees *et al.* [67] proposed a hybrid of the artificial bee colony (ABC) and sine cosine algorithm (SCA) for MLT image segmentation. The SCA is employed as a local search for the ABC to enhance its performance. The hybrid model was applied for MLT using several images and showed good performances compared to several existing MH methods. In [68], an MLT method based on fuzzy entropy and a hybrid of the salp swarm optimizer (SSO) and the MFO was proposed. It was evaluated using different images, and it showed better performance compared to individual optimization algorithms. Furthermore, a hybrid of gravitational search algorithm and GA was proposed by [69] for MLT image segmentation using the entropy fitness function. Also, a hybrid of the spherical search optimizer (SSO) and SCA is proposed by [70]. Fuzzy entropy is applied as the fitness function. The proposed model also confirms its performance using different images and by comparing it to several state-of-art models.

Moreover, MLT also has been used for medical image segmentation; for example, Li *et al.* [34] proposed a dynamic-context cooperative quantum-behaved PSO based on MLT for CT image segmentation. They used six different CT images to test the performance of the improved PSO, which showed significant performance. Also, Li *et al.* [71] proposed an MLT for medical image segmentation based on a partitioned and cooperative quantum-behaved PSO. They test the improved PSO with four stomach CT images, and they compared it to two modified PSO algorithms. Chatterjee *et al.* [35] proposed an MLT method with three-level thresholding for human head CT image segmentation. They applied an improved biogeography based optimization (BBO) and fuzzy entropy to segment fifteen CT images. The improved BBO was compared to PSO, GA, and it showed better performance. Also, in [36], an MLT method with PSO is applied for lung high-resolution CT image segmentation.

Panda *et al.* [37] proposed an MLT approach for brain MR image segmentation based on an evolutionary gray gradient algorithm (EGGA). They also applied an adaptive swallow swarm optimization (ASSO) algorithm to optimize the fitness function. They used twenty-five MR images to evaluate the ASSO, which showed better performance than the original SSO. Wang *et al.* [72] presented an MLT approach to segment medical images based on an improved FPA algorithm. They applied Otsu’s as an objective function. They used Eight CT images to evaluate the proposed approach, which outperformed several MH algorithms, including the original FPA, PSO, GA, and DE. Mostafa *et al.* [20] applied the WOA for liver MRI image segmentation. They used several measures to evaluate the WOA, including structural similarity index measure (SSIM) and similarity index (SI). The WOA achieved high accuracy rates in both measures. Ladgham *et al.* [38] proposed an enhanced Shuffled Frog Leaping Algorithm (SFLA) for MR brain image segmentation. They compared it to the original SFLA and the GA, and it showed significant performance. Raja *et al.* [39] applied the bat algorithm (BA) to enhance the segmentation process of the MRI images. In [73], the FA is used to optimize SVM classifier to classify lung CT images. Also, the gray wolf optimizer (GWO) was used with the artificial neural network (ANN) to classify MRI images [74]. Also, in [75] the FA is applied for brain MRI segmentation.

## III. Methodology

### A. Problem Definition

The problem formulation of MLT is presented in this section. Assume we have a gray-scale image $I$, which has $K+1$ classes. To divide a given image $I$ into classes, the values of $k$ thresholds $\{t_{k}, k=1,2,K\}$ are needed, which can be defined as:

\begin{align*} C_{0}=&\{I_{ij} \mid 0 \leq I_{ij} \leq t_{1}- 1\}, \\ C_{1}=&\{I_{ij} \mid t_{1} \leq I_{ij} \leq t_{2}- 1\}, \\&\ldots \\ C_{K}=&\{I_{ij} \mid t_{K} \leq I_{ij} \leq L- 1\}\tag{1}\end{align*}

where $L$ represents the maximum gray levels, $C_{K}$ is the $k$th class of the image, $t_{k}$ is the $k$-th threshold, and $I_{ij}$ represents gray levels at $(i,j)$-th pixel. Where the problem of the MLT can be defined as a maximization problem which is applied to find an optimal threshold value as:

\begin{equation*} t_{1}^{*},t_{2}^{*},\ldots,t_{K}^{*}=\arg \max _{t_{1},\ldots,t_{K}} Fit({t_{1},\ldots,t_{K}})\tag{2}\end{equation*}

where $Fit$ is the objective function. Here, the Fuzzy entropy [14] is applied as an objective function. Fuzzy entropy is a popular technology [76]–[78], which has been applied in many multi-level threshold segmentation applications, such as color images [79], brain tumor images [80], MRI images [81] and others [82], [83]. It can be defined as:

\begin{align*} Fit({t_{1},\ldots,t_{K}})=&\sum _{k=1}^{K} H_{i} \tag{3}\\ H_{k}=&-\sum _{i=0}^{L-1}\frac {p_{i}\times \mu _{k}(i)}{P_{k}} \times ln\left({\frac {p_{i}\times \mu _{k}(i)}{P_{k}}}\right),\qquad \tag{4}\\ P_{k}=&\sum _{i=0}^{L-1}{p_{i}\times \mu _{k}(i)} \tag{5}\\ \mu _{1}(l)=&\begin{cases} 1 & l\leq a_{1} \\ \dfrac {l-c_{1}}{a_{1}-c_{1}} & a_{1} \leq l\leq c_{1} \\ 0 & l> c_{1} \\ \end{cases} \tag{6}\\ \mu _{K}(l)=&\begin{cases} 1 & l\leq a_{K-1} \\ \dfrac {l-a_{K}}{c_{K}-a_{K}} & a_{K-1} < l\leq c_{K-1} \\ 0 & l> c_{K-1} \\ \end{cases}\tag{7}\end{align*}

In Eq. (7), $p_{i}$ is the probability distribution which is computed as $p_{i}=h(i)/N_{p}$ ( $0 < i < L-1$); where $h(i)$ and $N_{p}$ are the number of pixels for the corresponding gray level $L$ and total number of pixels in $I$.

$a_{1},c_{1},\ldots.,a_{k-1},c_{k-1}$ are the fuzzy parameters, where $0\leq a_{1}\leq c_{1}\leq \ldots \leq a_{K-1} \leq c_{K-1}$. Then $t_{1}=\frac {a_{1}+c_{1}}{2}, t_{2}=\frac {a_{2}+c_{2}}{2},\ldots, t_{K-1}=\frac {a_{K-1}+c_{K-1}}{2}$.

## IV. Marine Predators Algorithm

Faramarzi *et al.* [40] introduced a novel meta-heuristic (MH) optimization algorithm inspired by the prey and predator characteristics in nature. The developed algorithm named Marine Predators Algorithm (MPA). The creatures usually aimed to find their foods and continuously searching for them. Hence, the predator is searching for its food as well as the prey is looking for its food. Based on this concept, Faramarzi *et al.* [40] designed the MPA algorithm.

At the first stage, the predator/prey stats discovering the search space to detect their food location, then they convergence for its position to catch it from this principle the MHs are established. MPA started by discovering the search space via a random set of solutions as an initialization. Then those solutions are updates based on the mainframe of the technique.

The initialization stage can be given based on the search space boundaries as below;

\begin{align*}&\hspace {-.5pc}U_{ij}=lb_{j}+r_{1}\times (ub_{j}-lb_{j}), \\& \qquad \qquad \qquad\qquad  \displaystyle {j=1,2,\ldots,D, \, i=1,2,\ldots,N  }\tag{8}\end{align*}

where the $lb_{j}$ and $ub_{j}$ are the lower and upper boundaries in the search space at dimension $j$, $r_{1}$ is a random number withdrawn from a uniform distribution in the interval of [0, 1].

As mentioned earlier both the prey and predator are searching for their foods; therefore, there are two main matrices should be defined, the Elite matrix (matrix of the fittest predators) and the prey matrix that can be defined as below:

\begin{align*} Elite=\left [{\begin{array}{cccc} U_{11}^{1}&\quad U_{12}^{1}&\quad \ldots &\quad U_{1d}^{1}\\ U_{21}^{1}&\quad U_{22}^{1}&\quad \ldots &\quad U_{2d}^{1}\\ \ldots &\quad \ldots &\quad \ldots &\quad \ldots \\ U_{n1}^{1}&\quad U_{n2}^{1}&\quad \ldots &\quad U_{nd}^{1}\\ \end{array}}\right], \\ \, U=\left [{\begin{array}{cccc} U_{11}&\quad U_{12}&\quad \ldots &\quad U_{1d}\\ U_{21}&\quad U_{22}&\quad \ldots &\quad U_{2d}\\ \ldots &\quad \ldots &\quad \ldots &\quad \ldots \\ U_{n1}&\quad U_{n2}&\quad \ldots &\quad U_{nd}\\ \end{array}}\right],\tag{9}\end{align*}

where $U_{ij}$ refers to the value of the $i$th solution at $j$th dimension. To catch the global optimum solutions, the initial solutions should be modified based on the main structure of the MPA. MPA maintains three stages for adjusting the initial solutions. The followed steps have relied on the velocity ration between prey and predator. The first phase can be regarded once the velocity ratio between predator and prey is high. In contrast, the unit and low-velocity rates are measurable for the second and third stages. Details of each step are addressed below.

### A. Stage 1: Exploration Phase (High-Velocity Ratio)

For the first third of the total number of iterations, i.e., $\frac {1}{3}t_{max}$) in MPA, the search agents start to discover the search space where the exploration stage is accomplished. The prey hurries to search for its food while the predator waits to monitor its motion. That is why the high-velocity ratio among the prey and predator is the primary feature of this stage. Accordingly, the prey location is modifying using the following equations.

\begin{align*} S_{i}=&R_{B} \bigotimes (Elite_{i}-R_{B}\bigotimes U_{i}),\quad i=1,2,\ldots,n\qquad \tag{10}\\ U_{i}=&U_{i}+P.R\bigotimes S_{i}\tag{11}\end{align*}

where $R\in [{0,1}]$ is a random vector withdrawn from a uniform distribution, and $P=0.5$ is a constant number. The symbol of $R_{B}$ refers to Brownian motion. $\bigotimes $ indicates the process of element-wise multiplications.

### B. Stage 2: Transition Among the Exploration and Exploitation (Unit Velocity Ratio)

After detecting the closest position for the foods, the prey/predator starts to exploit this location; therefore, this stage is considered as the transmission phase among the exploration and exploitation capabilities. This stage is the middle stage of the algorithm when $\frac {1}{3}t_{max} < t < \frac {2}{3}t_{max}$ where both the prey and predator move with the nearly same velocity. The predator follows Brownian motion while the prey follows the lévy flight sequentially Faramarzi *et al.* [40] divided the population for two halves and implemented Eqs. (13)–(14) to model the motion of the first half of the population and Eq. (15)–(16) for the second half as represented below.

\begin{align*} S_{i}=&R_{L} \bigotimes (Elite_{i}-R_{L}\bigotimes U_{i}),\quad i=1,2,\ldots,n72\qquad \tag{12}\\ U_{i}=&U_{i}+P.R\bigotimes S_{i}\tag{13}\end{align*}

where $R_{L}$ has random numbers that follow Lévy distribution. Eqs. (13)–(14) are applied to the first half of the agents that represents the exploitation. While the second half of the agents perform the following equations.

\begin{align*} S_{i}=&R_{B} \bigotimes (R_{B} \bigotimes Elite_{i}{-} U_{i}),\quad i=1,2,\ldots,n/2\qquad \tag{14}\\ U_{i}=&Elite_{i}+P.CF\bigotimes S_{i},\, CF=\left({1-\frac {t}{t_{max}} }\right)^{2\frac {t}{t_{max}}\big)}\tag{15}\end{align*}

where $CF$ is the parameter that controls the step size of movement for predator.

### C. Stage 3: Exploitation Stage (Low-Velocity Ratio)

This stage is the last stage in the optimization process as the predator exploits the detected location of the prey and move very fast to catch it. This stage executed on the last third of the iteration numbers ( $t>\frac {2}{3}t_{max}$) where the predator follows Lévy during updates its position based on the following formula:

\begin{align*} S_{i}=&R_{L} \bigotimes (R_{L} \bigotimes Elite_{i}- U_{i}),\quad i=1,2,\ldots,n\qquad \tag{16}\\ U_{i}=&Elite_{i}+P.CF\bigotimes S_{i},\, CF=\left({1-\frac {t}{t_{max}} }\right)^{2\frac {t}{t_{max}}\bigg)}\tag{17}\end{align*}

### D. Eddy Formation and Fish Aggregating Devices’ Effect (FADS)

In the purpose of avoiding the local optimum solutions, Faramarzi *et al.* [40] considered the external impacts from the environment such as the eddy formation or Fish Aggregating Devices (FADs) effects that can be mathematically formulated as below:

\begin{align*} U_{i}=\begin{cases} U_{i}+CF [U_{min}+R \bigotimes (UDif)]\bigotimes W & r_{5} < FAD \\ U_{i}+[FAD(1-r)+r](U_{r1}-U_{r2}) & r_{5} > FAD\\ \end{cases} \\\tag{18}\end{align*}

In Eq. (19), $UDif=U_{max}-U_{min}\,\,FAD=0.2$, and $W$ is a binary solution 0 or 1 that corresponded to random solutions. If the random solution is less than 0.2, it converted to 0 while the random solution becomes 1 when the solutions are greater than 0.2. The symbol of $r\in [{0,1}]$ represents a random number. $r_{1}$ and $r_{2}$ are the random index of the prey.

### E. Marine Memory

The marine predators have a feature that helps in catching the optimal solution very fast and avoid the local solutions is that memorizing the location of the high production foraging. Faramarzi *et al.* [40] implement this feature in his algorithm via saving the previous best solutions of a prior iteration and compared with the current ones. The solutions are modified based on the best one during the comparison stage. The pseudo-code of MPA is presented below 1.

Algorithm 1 **Steps of MPA**
1: Set the initial value for a set of $N$ agents $U$.
2: **while** termination criteria are not met **do**
3: Compute the fitness value and build in Elite matrix.
4: **if** $t < t_{max}/3$ **then**
5: Update value of agent using Eq. (12).
6: **else if** $t_{max}/3 < t < 2 \times t_{max}/3$ **then**
7: For the first half of the agents ( $i=1,\ldots,N/2$).
8: Update value of agent using Eq. (14).
9: For the second half of the agents ( $i=1,\ldots,N/2$).
10: Update value of agent using Eq. (16).
11: **else if** $t>2 \times t_{max}/3$ **then**
12: Update value of agent using Eq. (18).
13: **end if**
14: Using FADs effect and Eq. (19) to update current agent.
15: Update memory and Elite.
16: **end while**

### F. Moth-Flame Optimizer

Mirjalili [84] proposed the moth-flam optimizer based on the navigation behavior of moths at night that known by transverse orientation methodology. The moth utilized a fixed angle with the moon during its fly that helps it to reach for its goal, especially when the light is far. In contrast, the moths follow spirally flying around the near source of the light. Mirjalili [84] addressed another feature in MFO algorithm as the moths search around the flame and continually update this flame; therefore, not only the moths are the solutions but also the flames. Both the moths and flames locations are modified across the iterations number whereas with following diff rent control equations. The moths are the search agents, while flames are the best obtained moths location so far. Mirjalili [84] modeled these behaviors for mathematical equations to form his techniques MFO algorithm. MFO as all the MHs starts with random solutions, initialization phase then the solutions are modified based on the main equations of the algorithm, and at the end, the algorithm is stopped based on its termination criteria as presented as follows [84]:

\begin{equation*} MFO=(Init,Main,Ter),\tag{19}\end{equation*}

where $Init$ is the initialization phase that is responsible for creating the first random solutions as bellow

\begin{align*} U(i,j)=&(ub(i)-lb(i))* rand()+lb(i), \tag{20}\\[5pt] OM=&SAE=FitnessFunction(U),\tag{21}\end{align*}

where $lb$, $ub$ are the lower and upper bounds of the variables, respectively.

The $Main$ function in Eq. 20 includes the main structure of the MFO where the MFO motions are modeled and updated based on the logarithmic spiral function to emulate the transverse orientation of moths as below [84]:

\begin{equation*} S(U_{i},F_{j})= | F_{j}- U_{i}| e^{bd} cos(2 \pi d)+F_{j}, \tag{22}\end{equation*}

where $U_{i}$, $F_{j}$ refer to the $i$-th, $j$-th moth and flame, respectively. The symbol of $S $ denotes the spiral function, $b$ is a control parameter for the shape of the logarithmic spiral, and $d~\in ~[r,1]$ is a random number. The $r$ values are linearly decreased from −1 to −2 in order to accelerate the convergence speed of MFO where the smaller $d$, the closer the distance to the flame.

In MFO, Mirjalili [84] adaptively update the number of flames across the iterations to balance between the diversification and intensification phases, as in equation. (24). The equations reveal on decreasing for the number of the flames across the iteration numbers thereby at the last iterations the moths update their locations only with respect to the best flame [84]:

\begin{equation*} flame no= round\left ({N_{f}-t* \frac {N_{f}-1}{t_{max}}}\right),\tag{23}\end{equation*}

where $t$ is the current number of iteration, $N_{f}$ is the maximum number of flames, and $t_{max}$ is the maximum number of iterations.

The final steps of the MFO are illustrated in Algorithm 2.

Algorithm 2 **Steps of MFO**
1: Producing the initial population $U$.
2: set $t=1$.
3: **while** ( $t < t_{max}$) **do**
4: calculate objective value for $U_{i}$.
5: Sort $U$ and determine the best solution ( $U_{b}$).
6: Using Eq. (24) to update $Flames_{N}$.
7: **for** $i=1:N$ **do**
8: Using Eq. (23) to update $U_{i}$.
9: **end for**
10: **end while**
11: Return $U_{b}$.

## V. Proposed Image Segmentation Method

**FIGURE 1.**
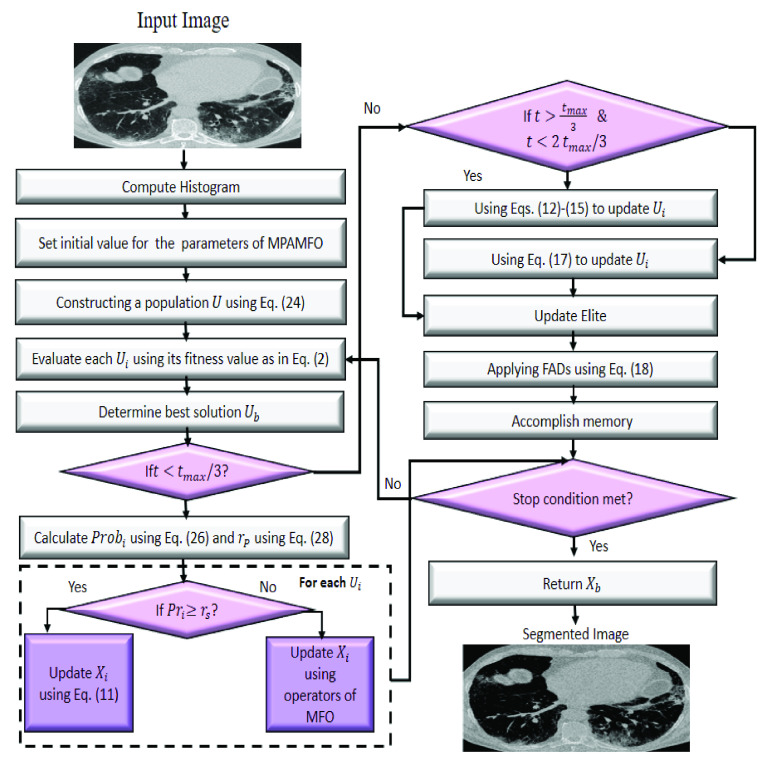
The steps of MPAMFO approach.

In this section, the steps of the proposed multi-level threshold approach are introduced, as in Figure 1. The developed model depends on improving the performance of the Marine Predators Algorithm (MPA) using the operators of moth-flame optimization (MFO). This achieved by using the operators of MFO to make the agents are competitive during the exploration phase since it has been found that the main weakness of MPA is its ability to explore the search space. In general, the modified MPA is called MPAMFO starts by setting initial value for a set of $N$ agents $X$. This performed by using the following equation:

\begin{equation*} U_{i,j}\!=\!I_{min,j}\!+\!r_{1}\times (I_{max,j}\!-\!I_{min,j}),\quad j =1, 2,\ldots, D,\tag{24}\end{equation*}

In Eq. 25, $I_{min,j}$ and $I_{max,j}$ are the minimum and maximum gray value of $I$ at $j$th dimension, respectively. In addition, $D=2K$ where $K$ is the threshold level that needs to segment the image at it. The next process is to compute the fitness value $Fit$ for each agent using Eq. (2). Then determine the agent that has the best $Fit$ and used it as best agent $U_{b}$. Thereafter, the agent will update their values using either the operators of exploration or exploitation, as discussed in section IV. However, during the exploration, the probability ( $Pr_{i}$) of each agent depends on its fitness value, is computed using Eq. (26).

\begin{equation*} Pr_{i}=\frac {Fit_{i}}{\sum _{i=1}^{N}Fit_{i}}\tag{25}\end{equation*}

Thereafter, the agents in the exploration phase are updated using the following equation:

\begin{align*} U_{i}=\begin{cases} operators\, of\, MPA & Pr_{i} >r1 \\ operators\, of\, MFO & otherwise \\ \end{cases}\tag{26}\end{align*}

where

\begin{align*} r_{s}\!=\!min(Pr_{i})\!+\!rand\!\times \! (max(Pr_{i})\!-\!min(Pr_{i})), \, rand\!\in \![{0,1}] \\\tag{27}\end{align*}

From Eq. (27), when the value of $Pr\geq r1$, then the operators of MPA are used, otherwise the operators of MFO are used. In addition, we applied Eq. (28) to avoid the problem of fixing it to a specified value, so the value of $r1$ is automatically updated depends on the value of $Pr$.

From Eq. (27), when the value of $Pr\geq r1$, then the operators of MPA are used, otherwise the operators of MFO are used. In addition, we applied Eq. (28) to avoid the problem of fixing it to a specific value, so the value of $r1$ is automatically updated depends on the value of $Pr$.

The next step is to check the stop conditions when they are met, then the best solution is considered the output. From the value of $U_{b}$ that refers to the fuzzy parameters are used to form the threshold value as $t_{k}=\frac {U_{b}^{k}+U_{b}^{k+1}}{2}$, where $k=1:2:K-1$.

Computational Complexity: The computational complexity of MPAMFO depends on some factors such as number of fitness evaluation $EF$, number of solutions $N$, total number of iterations $t_{max}$, and the number of thresholds $K$. In addition, since MFO is one of main component of MPAMFO so its complexity also influence on the total complexity of MPAMFO. So, the complexity $O\left ({{MPAMFO} }\right)$ of MPAMFO formulated as: In Best case:

\begin{equation*} O\left ({N\times {t_{max}\left ({{(N+1)K \!+\! EF}\!+\!(N\!-\!K_{p})\!\times \! log(N)}\right)} }\right)\tag{28}\end{equation*}

In worst case:

\begin{equation*} O\left ({N\times {t_{max}\left ({{(N+1)K + EF}+(N-K_{p})\times N^{2}}\right)} }\right)\tag{29}\end{equation*}

where $K_{p}$ denotes the number of solution that using the operators of MPA to update their values.

## VI. Experiments and Results

In this section, two experiments are used to evaluate the performance of the MPAMFO. It is compared with eight algorithms namely, original MPA, harris hawks optimization (HHO) [85], cuckoo search (CS) [86], grey wolf optimization (GWO) [87], grasshopper optimization algorithm (GOA) [88], spherical search optimization (SSO) [89], particle swarm optimization (PSO) [90], and moth-flame optimization (MFO) [84]. Besides, using two sets of images. These algorithms established their quality as MLT image segmentation methods in literature.

### A. Performance Measures

In order to assess the quality of the segmented image, we used a set of performance metrics, including Peak Signal-to-Noise Ratio (PSNR) [91], [92], and the Structural Similarity Index (SSIM) [93]. PSNR and SSIM can be defined as:

\begin{align*} PSNR=&20log_{10}\left({\frac {255}{RMSE}}\right), \\ RMSE=&\sqrt {\frac {\sum _{i=1}^{N_{r}} \sum _{j=1}^{N_{c}} (I_{i,j}-I_{S}{i,j})^{2}}{N_{r} \times N_{c}}}\tag{30}\end{align*}

here, the $RMSE$ is the root mean-squared error. $I$ and $I_{S}$ refer to the original and segmented images with the size $N_{r}\times N_{c}$, respectively.

\begin{equation*} SSIM(I,I_{S})=\frac {(2~\mu _{I} \mu _{I_{S}} +c_{1})(2\sigma _{I,I_{S}} +c_{2})}{(\mu _{I}^{2} +\mu _{I_{S}}^{2} +c_{1})(\sigma _{I}^{2}+\sigma _{I_{S}}^{2} +c_{2})}\tag{31}\end{equation*}

$\mu _{I}$( $\sigma _{I}$) and $\mu _{I_{S}}$ ( $\sigma _{I_{S}}$) refers to the images’ mean intensity (standard deviation) of $I$ and $I_{S}$, respectively. The $\sigma _{I, I_{S}}$ is the covariance of $I$ and $I_{S}$. The values of the constants $c_{1}$ and $c_{2}$ are set to 6.5025 and 58.52252, respectively following [61]. Furthermore, we use the fitness value to evaluate the quality of threshold values; also, we use the CPU time for each algorithm.

### B. Parameters Setting

Table 1 lists the parameter settings for the algorithms that are applied in the following experiments. In addition, the general parameters are set as follows. The population number is set to 20, and the total number of iteration is 100. More so, 30 independent runs were performed for each method.

**TABLE 1:** Parameters Setting

Algorithm	Parameters setting
MPA	$FADs = 0.2, \,\,P = 0.5, \,\,\beta = 1.5$
MPAMFO	$FADs = 0.2, \,\,P = 0.5, \,\,\beta = 1.5. \,\,b=1 $
HHO	$E \in [{0, 2}]$
CS	pa=0.25
GWO	$a \in [{2, 0}]$
GOA	$c_{max} = 1$, $c_{min} = 0.00004$
SSO	$\omega \in [0,2\pi], ~F\in [{0,1}], ~\theta \in [0,\pi]$
PSO	$w_{Max} = 0.9,\,\,w_{Min} = 0.2, \,\,C1 = 2, \,\,C2 = 2$
MFO	$b=2$

### C. First Experiment

**FIGURE 2.**
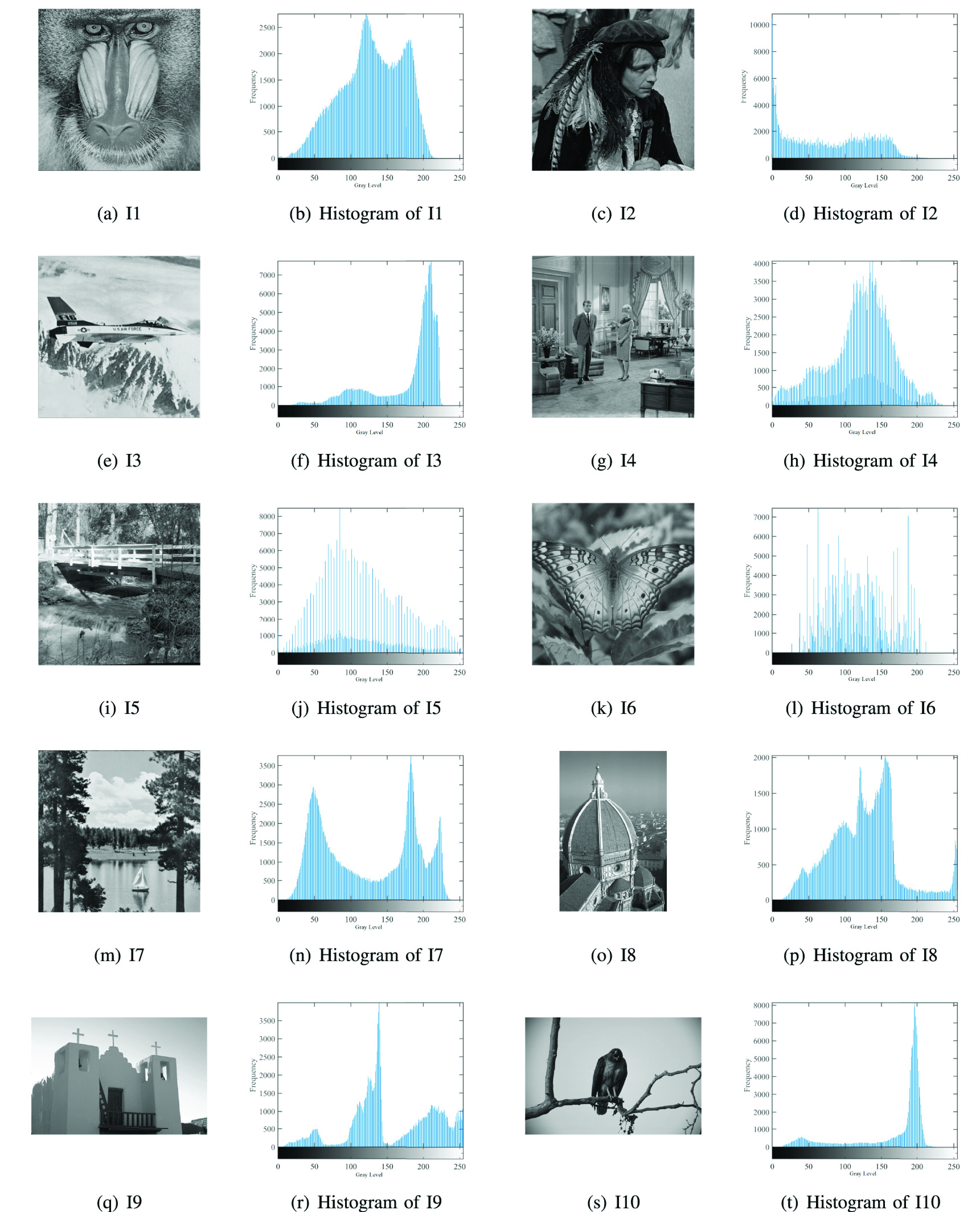
Histograms and original images.

In this experiment, a set of ten images has been used to compute the quality of the proposed method. As can we observed from Figure 2, these images have different characteristics according to their histogram. The MPAMFO aims to segment those images at different levels of thresholds, these levels equal to 6, 8, 15, 17, 19, and 25.

**FIGURE 3.**
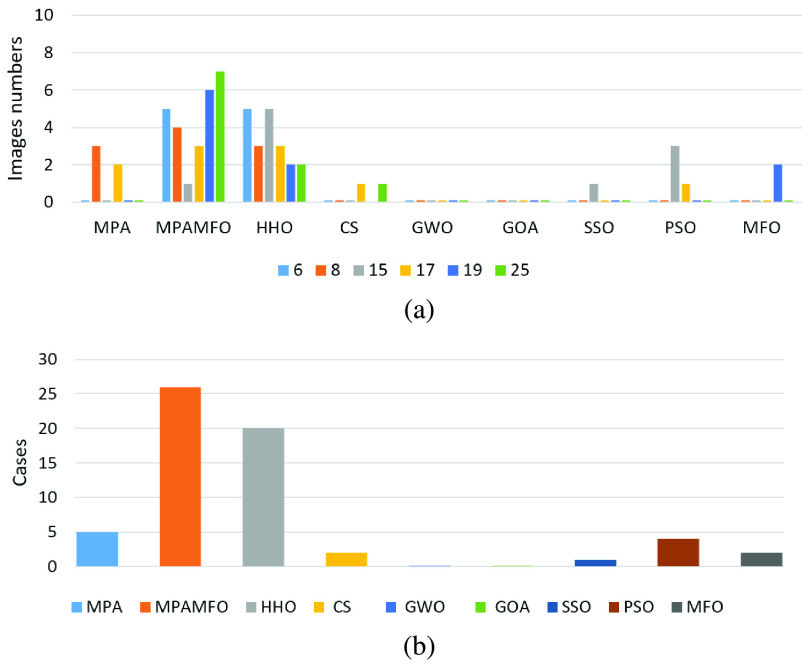
Summary of the PSNR results for the first experiment. (a) illustrates the performance of each algorithm at thresholds levels. (b) illustrates the numbers of the best cases obtained by each algorithm.

**FIGURE 4.**
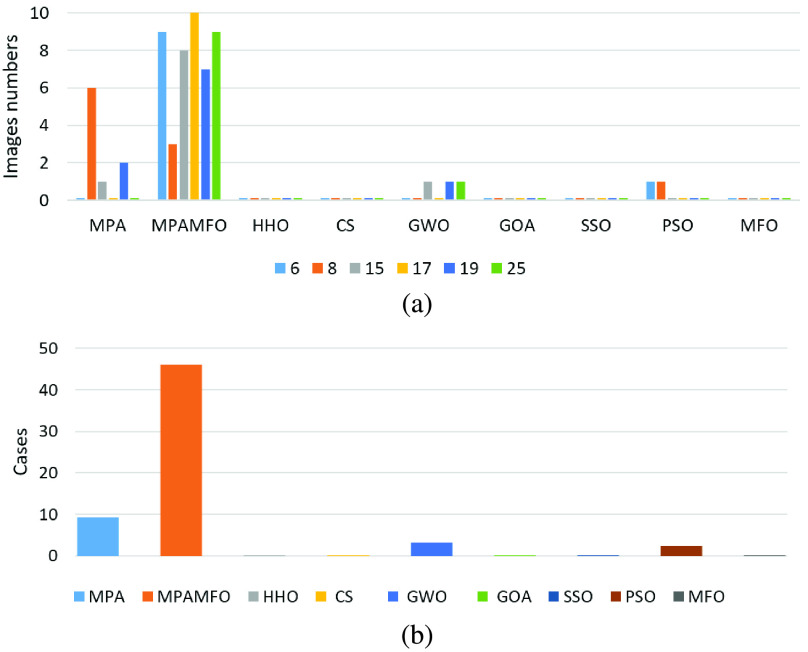
Summary of the SSIM results for the first experiment. (a) illustrates the performance of each algorithm at thresholds levels. (b) illustrates the numbers of the best cases obtained by each algorithm.

**FIGURE 5.**
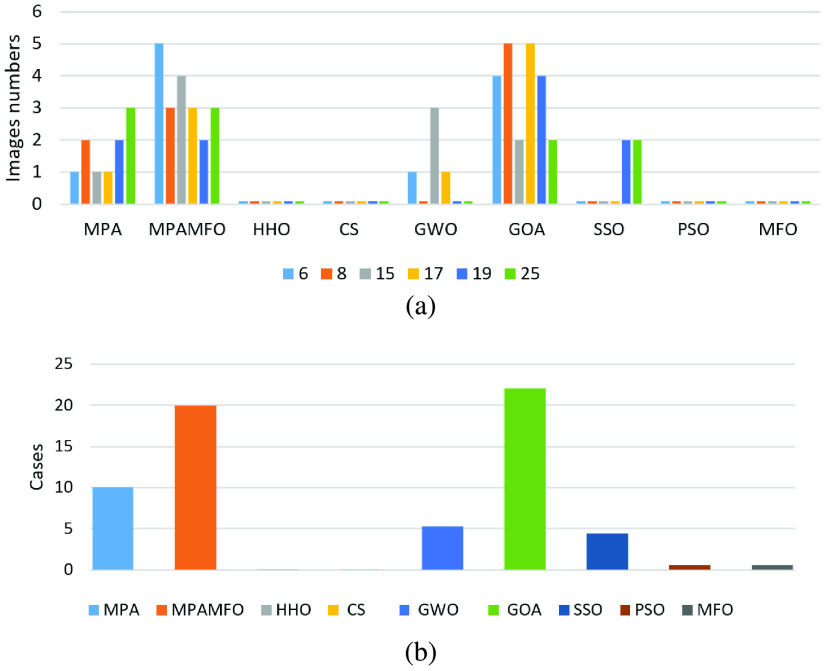
Summary of the fitness value results for the first experiment. (a) illustrates the performance of each algorithm at thresholds levels. (b) illustrates the numbers of the best cases obtained by each algorithm.

The results are introduced in Tables 2–4 and Figures 3–5. Table 2 shows the results of the PSNR measure for all images. In detail, at level 6, the performance of the MPAMFO is similar to the HHO algorithm; they achieved the best PSNR values in 5 images for each one followed by MPA, SSO, CS, GWO, PSO, and MFO, respectively. At level 8, the MPAMFO achieved the best PSNR in 4 images and is ranked first, followed by MPA, HHO, PSO, SSO, MFO, GWO, and CS, respectively. At level 15, the HHO algorithm obtained the highest PSNR value in 5 images followed by the MPAMFO. The PSO, MFO, and MPA achieved the third, forth, and fifth rank. However, the MPAMFO does not obtain the first rank, its performance is very close to the HHO algorithm in most of the images. At level 17, both MPAMFO and HHO algorithms obtained the highest PSNR value in 3 images followed by the PSO, CS, and MFO. At levels 19 and 25, the MPAMFO obtained the best PSNR values in 60% and 70%, respectively, of all images. The HHO algorithm came in the second rank with only two images for each level. The CS is ranked third, followed by PSO, SSO, MFO, and MPA. Whereas, the GOA algorithm recorded the worst results at all levels.

**TABLE 2:** PSNR Results for the First Experiments

Level (K)	Image	MPA	MPAMFO	HHO	CS	GWO	GOA	SSO	PSO	MFO
6	I1	14.002	16.859	15.233	14.254	14.123	13.562	14.598	11.847	10.774
	I2	16.250	16.244	16.563	15.881	15.612	15.455	15.964	12.495	12.476
	I3	10.039	14.984	15.425	12.881	12.605	11.330	13.483	10.720	10.763
	I4	14.761	16.697	16.413	16.211	16.331	16.025	16.010	11.028	10.776
	I5	12.703	15.329	13.730	11.666	11.903	10.880	12.563	10.806	10.490
	I6	13.417	13.233	14.552	11.924	12.183	11.507	12.603	10.955	10.374
	I7	11.744	14.805	14.062	11.983	11.822	11.687	12.334	12.451	11.852
	I8	13.520	15.269	14.906	14.489	14.019	13.397	14.172	10.551	10.787
	I9	11.096	13.121	13.561	10.151	10.599	9.386	10.191	11.054	9.928
	I10	10.716	15.815	16.429	14.212	14.424	13.073	14.702	13.122	12.049
8	I1	17.684	18.747	17.980	18.151	17.706	17.051	18.162	17.989	16.840
	I2	20.117	21.272	18.227	16.894	16.539	15.643	17.067	17.825	15.266
	I3	11.852	16.635	17.173	15.720	15.964	14.909	16.561	16.278	16.175
	I4	18.522	18.419	17.249	17.698	17.064	16.777	17.736	18.241	17.185
	I5	16.222	17.311	16.168	16.013	16.157	15.748	15.723	16.431	16.054
	I6	18.006	17.873	17.727	15.184	15.585	14.066	15.741	17.116	16.225
	I7	14.614	16.541	16.842	15.996	15.544	15.139	16.239	16.022	15.704
	I8	17.222	17.029	16.336	15.153	16.899	14.709	15.062	16.833	16.423
	I9	12.830	16.898	16.934	15.504	15.424	14.237	15.663	14.987	16.155
	I10	12.581	19.909	19.079	19.108	19.316	18.182	18.338	17.830	17.730
15	I1	22.285	22.327	23.361	23.013	21.509	20.835	22.868	21.847	20.842
	I2	23.519	23.664	23.141	22.437	22.187	20.035	22.457	23.379	20.748
	I3	16.773	17.613	22.895	21.528	19.667	19.299	21.927	23.026	17.105
	I4	22.004	21.866	22.179	21.667	21.685	19.882	22.547	22.977	21.057
	I5	21.389	21.348	22.851	21.165	21.295	18.609	21.149	20.250	20.888
	I6	21.956	22.574	23.204	21.151	20.510	17.751	21.951	23.115	22.510
	I7	20.257	20.146	21.458	21.324	20.229	18.422	21.547	19.913	20.495
	I8	22.289	22.282	22.649	21.823	21.299	18.722	21.601	21.748	22.505
	I9	18.935	21.348	21.457	20.969	18.096	17.775	19.950	19.989	21.206
	I10	19.707	22.813	23.306	21.459	21.467	19.492	21.416	24.165	20.719
17	I1	23.596	24.544	24.427	24.529	23.075	22.315	24.233	23.525	21.207
	I2	24.587	24.493	24.081	24.146	24.048	20.855	23.838	23.653	22.454
	I3	19.227	23.936	24.209	23.327	20.658	20.903	23.356	24.306	23.505
	I4	23.248	24.088	24.217	22.894	22.487	20.985	22.883	24.194	23.322
	I5	22.399	24.630	23.208	22.685	22.868	20.365	22.299	22.892	23.089
	I6	23.113	24.739	25.263	22.213	22.155	19.231	23.945	23.480	22.317
	I7	21.510	23.741	23.548	22.614	21.414	20.145	22.164	22.094	22.598
	I8	23.485	23.242	23.294	22.681	22.887	19.943	23.237	22.843	23.474
	I9	20.607	22.078	22.632	22.704	19.356	18.916	21.635	21.320	22.526
	I10	21.697	23.547	23.991	23.155	21.930	20.542	23.026	23.223	22.035
19	I1	24.517	26.348	25.449	25.236	24.251	23.077	25.151	24.320	24.370
	I2	25.521	25.914	25.311	25.350	24.971	22.273	24.569	25.250	24.647
	I3	20.620	26.781	25.517	24.743	21.786	21.583	25.124	24.976	23.371
	I4	24.561	24.649	23.939	23.709	23.913	21.438	23.342	23.916	23.451
	I5	23.384	25.425	24.976	24.154	23.857	21.752	23.178	24.064	23.724
	I6	24.401	25.414	26.355	24.851	23.623	20.327	24.041	24.136	24.216
	I7	23.339	24.646	24.137	24.532	22.666	21.274	24.273	24.346	23.158
	I8	24.016	24.223	24.105	24.152	23.879	20.465	24.155	24.208	24.848
	I9	21.206	24.278	23.359	22.523	20.864	19.788	22.468	22.311	24.358
	I10	22.093	24.479	25.254	24.317	22.756	21.452	24.126	23.108	23.434
25	I1	26.755	28.696	27.710	27.401	26.732	25.759	27.409	28.313	26.519
	I2	27.586	28.751	27.851	28.227	28.058	26.214	27.747	27.481	27.394
	I3	24.424	28.127	28.267	26.803	23.930	23.908	27.446	27.545	26.903
	I4	26.553	29.200	27.601	26.752	26.257	24.955	26.336	28.649	27.207
	I5	26.168	27.172	26.954	27.395	26.906	24.826	26.330	27.115	26.562
	I6	26.884	27.747	28.624	26.745	27.180	23.776	28.320	27.276	25.356
	I7	25.663	28.684	27.453	27.406	25.971	24.731	26.792	26.051	25.698
	I8	26.673	28.266	27.085	27.203	26.709	24.640	26.669	27.115	26.163
	I9	24.804	27.881	26.439	26.565	24.435	23.307	25.730	27.285	25.832
	I10	26.179	28.727	28.032	27.664	25.956	24.661	27.600	26.258	25.700

**TABLE 3:** SSIM Results for the First Experiments

Level (K)	Image	MPA	MPAMFO	HHO	CS	GWO	GOA	SSO	PSO	MFO
6	I1	0.5058	0.6032	0.5872	0.5235	0.5103	0.4897	0.5391	0.4156	0.3673
	I2	0.4192	0.4745	0.4585	0.4040	0.4023	0.3849	0.4089	0.2248	0.2530
	I3	0.5983	0.6828	0.6668	0.6162	0.6072	0.6125	0.6366	0.6113	0.6187
	I4	0.4835	0.5894	0.5734	0.5448	0.5513	0.5386	0.5351	0.2871	0.2726
	I5	0.3767	0.4511	0.4351	0.2994	0.3153	0.2469	0.3557	0.2370	0.2260
	I6	0.4175	0.5022	0.4862	0.3415	0.3617	0.3087	0.3845	0.2679	0.2366
	I7	0.4291	0.5485	0.5325	0.4191	0.4188	0.3957	0.4297	0.4016	0.3934
	I8	0.5447	0.6422	0.6262	0.5921	0.5727	0.5410	0.5790	0.3885	0.4090
	I9	0.7189	0.7760	0.7600	0.5780	0.7024	0.5644	0.5437	0.5829	0.5218
	I10	0.7303	0.7387	0.7227	0.6603	0.6614	0.6270	0.6831	0.7687	0.7444
8	I1	0.7104	0.7252	0.7092	0.7146	0.7044	0.6805	0.7059	0.6943	0.6371
	I2	0.5863	0.5495	0.5335	0.4540	0.4567	0.4037	0.4644	0.4283	0.4812
	I3	0.7009	0.7957	0.7797	0.7611	0.7528	0.7520	0.7764	0.7745	0.7772
	I4	0.6586	0.6186	0.6026	0.6005	0.5889	0.5731	0.6048	0.6188	0.5839
	I5	0.6353	0.5817	0.5657	0.5522	0.5653	0.5366	0.5337	0.5704	0.5540
	I6	0.6475	0.6369	0.6209	0.5114	0.5342	0.4460	0.5323	0.5842	0.5334
	I7	0.6379	0.6339	0.6179	0.5850	0.5685	0.5367	0.5896	0.5552	0.5526
	I8	0.7154	0.7142	0.6982	0.6480	0.7071	0.6364	0.6336	0.6730	0.6679
	I9	0.7721	0.8313	0.8153	0.8056	0.8046	0.7806	0.8057	0.7995	0.8106
	I10	0.8199	0.7925	0.7765	0.7771	0.7632	0.7384	0.7698	0.8345	0.8218
15	I1	0.8352	0.8685	0.8525	0.8378	0.8128	0.7950	0.8357	0.8076	0.7880
	I2	0.7222	0.7299	0.7139	0.6742	0.7036	0.5865	0.6641	0.6523	0.5914
	I3	0.7897	0.8697	0.8537	0.8541	0.8498	0.8265	0.8465	0.8287	0.7899
	I4	0.7622	0.7760	0.7600	0.7395	0.7485	0.6808	0.7603	0.7743	0.7171
	I5	0.7980	0.8363	0.8203	0.7627	0.7845	0.6729	0.7626	0.7339	0.7547
	I6	0.7568	0.8078	0.7918	0.7408	0.7220	0.6174	0.7558	0.7723	0.7569
	I7	0.7858	0.7841	0.7681	0.7676	0.7929	0.6461	0.7642	0.6852	0.6896
	I8	0.8481	0.8554	0.8394	0.8248	0.8354	0.7664	0.8259	0.8275	0.8377
	I9	0.8676	0.8864	0.8704	0.8489	0.8542	0.8248	0.8321	0.8429	0.8438
	I10	0.9152	0.8937	0.8777	0.8620	0.8462	0.8235	0.8452	0.8873	0.8336
17	I1	0.8572	0.8866	0.8706	0.8716	0.8434	0.8306	0.8642	0.8465	0.7954
	I2	0.7553	0.7619	0.7459	0.7345	0.7562	0.6155	0.7206	0.6523	0.6851
	I3	0.8267	0.8914	0.8754	0.8687	0.8666	0.8503	0.8719	0.8463	0.8096
	I4	0.7927	0.8250	0.8090	0.7736	0.7722	0.7264	0.7722	0.7997	0.7788
	I5	0.8327	0.8440	0.8280	0.8101	0.8275	0.7448	0.8011	0.8201	0.8231
	I6	0.7860	0.8571	0.8411	0.7773	0.7747	0.6767	0.8048	0.7836	0.7532
	I7	0.7987	0.8256	0.8096	0.7876	0.8193	0.7288	0.7812	0.7550	0.7745
	I8	0.8704	0.8725	0.8565	0.8414	0.8635	0.7922	0.8515	0.8405	0.8517
	I9	0.8728	0.8899	0.8739	0.8592	0.8549	0.8308	0.8550	0.8591	0.8528
	I10	0.9240	0.9140	0.8980	0.8850	0.8550	0.8533	0.8783	0.8783	0.8610
19	I1	0.8761	0.9054	0.8894	0.8830	0.8653	0.8474	0.8806	0.8599	0.8695
	I2	0.7815	0.7965	0.7805	0.7644	0.7881	0.6684	0.7417	0.7400	0.7209
	I3	0.8384	0.9053	0.8893	0.8762	0.8741	0.8577	0.8800	0.8401	0.8353
	I4	0.8236	0.8199	0.8039	0.7928	0.8019	0.7343	0.7856	0.8036	0.7897
	I5	0.8438	0.8866	0.8706	0.8509	0.8525	0.7876	0.8245	0.8503	0.8380
	I6	0.8147	0.8795	0.8635	0.8361	0.8093	0.7149	0.8149	0.7970	0.7945
	I7	0.8303	0.8351	0.8191	0.8339	0.8400	0.7615	0.8206	0.8147	0.7712
	I8	0.8784	0.8853	0.8693	0.8766	0.8809	0.8065	0.8693	0.8677	0.8736
	I9	0.8833	0.8902	0.8742	0.8703	0.8711	0.8372	0.8699	0.8686	0.8783
	I10	0.9283	0.9168	0.9008	0.9050	0.8870	0.8703	0.8788	0.8959	0.8820
25	I1	0.9109	0.9381	0.9221	0.9151	0.9058	0.8951	0.9145	0.9320	0.9046
	I2	0.8239	0.8554	0.8394	0.8372	0.8647	0.7923	0.8200	0.8106	0.8202
	I3	0.8831	0.9221	0.9061	0.9041	0.9014	0.8830	0.8980	0.8916	0.8720
	I4	0.8593	0.8944	0.8784	0.8606	0.8544	0.8226	0.8518	0.8885	0.8678
	I5	0.9037	0.9235	0.9075	0.9126	0.9111	0.8664	0.8937	0.9106	0.8944
	I6	0.8650	0.9142	0.8982	0.8747	0.8864	0.8223	0.8918	0.8704	0.8317
	I7	0.8658	0.9016	0.8856	0.8798	0.8777	0.8439	0.8702	0.8571	0.8569
	I8	0.9142	0.9272	0.9112	0.9118	0.9145	0.8838	0.9036	0.9233	0.9000
	I9	0.9016	0.9166	0.9006	0.9035	0.8934	0.8740	0.8932	0.9112	0.8894
	I10	0.9173	0.9471	0.9311	0.9242	0.9223	0.9025	0.9262	0.9042	0.8845

**TABLE 4:** Results of the Fitness Function Value for All Algorithms

Level (K)	Image	MPA	MPAMFO	HHO	CS	GWO	GOA	SSO	PSO	MFO
6	I1	17.54	17.54	17.43	17.52	17.53	17.54	17.46	17.19	17.10
	I2	17.54	17.54	17.17	17.29	17.29	17.32	17.27	17.47	17.09
	I3	17.54	17.54	16.91	17.09	17.08	17.10	17.06	16.74	17.28
	I4	17.54	17.54	17.45	17.55	17.57	17.59	17.53	17.26	16.83
	I5	17.54	17.54	15.47	15.60	15.59	15.62	15.64	16.58	16.58
	I6	17.54	17.54	14.76	15.07	15.08	15.13	15.02	17.19	17.43
	I7	17.54	17.54	17.43	17.62	17.62	17.32	17.48	16.66	16.78
	I8	17.53	17.54	17.43	17.57	17.59	17.60	17.54	17.01	16.84
	I9	17.54	17.54	17.28	17.48	17.51	17.54	17.47	17.54	16.71
	I10	17.54	17.54	16.59	16.77	16.78	16.80	16.77	17.15	17.00
8	I1	20.85	20.85	20.62	20.77	20.82	20.84	20.69	20.50	20.80
	I2	20.85	20.85	20.55	20.78	20.82	20.91	20.69	20.00	20.36
	I3	20.85	20.84	20.28	20.44	20.45	20.54	20.38	19.92	20.28
	I4	20.86	20.85	20.73	20.91	20.95	21.01	20.85	20.11	20.42
	I5	20.85	20.86	18.17	18.26	18.32	18.38	18.26	20.54	20.32
	I6	20.85	20.84	17.05	17.39	17.43	17.50	17.28	19.98	20.37
	I7	20.84	20.85	20.69	20.87	20.91	20.95	20.83	19.89	20.80
	I8	20.85	20.85	20.63	20.87	20.84	20.99	20.86	20.00	20.32
	I9	20.84	20.85	20.64	20.98	21.04	21.06	20.99	20.16	19.92
	I10	20.84	20.85	19.82	19.98	20.02	20.06	19.92	20.73	20.51
15	I1	29.63	29.71	29.09	29.39	29.47	29.80	29.28	29.16	29.31
	I2	29.67	29.71	29.39	29.68	29.76	28.56	29.69	29.61	29.29
	I3	29.59	29.71	28.89	29.26	29.26	28.55	29.13	28.91	29.70
	I4	29.68	29.70	29.22	29.53	29.63	30.02	29.55	29.63	28.98
	I5	29.64	29.69	24.83	25.20	25.22	25.72	25.22	28.83	29.57
	I6	29.65	29.71	22.73	23.63	23.62	24.23	23.18	29.17	28.79
	I7	29.69	29.68	29.28	29.47	29.60	28.61	29.42	29.18	29.26
	I8	29.68	29.67	29.73	30.07	30.14	28.64	30.04	28.69	29.40
	I9	29.70	29.69	29.33	29.75	30.01	28.52	29.90	29.00	29.02
	I10	29.69	29.70	28.47	28.87	28.95	29.28	28.86	29.60	29.49
17	I1	32.31	32.37	31.80	31.96	31.94	31.08	31.84	32.24	31.84
	I2	32.31	32.30	32.11	32.39	32.43	33.01	32.42	31.91	31.38
	I3	32.30	32.33	31.46	31.79	31.79	32.43	31.70	32.04	31.45
	I4	32.28	32.28	31.75	32.13	32.14	32.76	32.18	32.07	31.78
	I5	32.33	32.36	26.62	27.16	27.21	27.74	27.22	31.95	32.26
	I6	32.28	32.36	24.19	25.28	25.29	26.12	24.64	31.72	32.24
	I7	32.33	32.29	31.83	32.11	32.19	32.63	32.10	31.70	31.57
	I8	32.34	32.30	32.28	32.68	32.71	33.34	32.66	32.11	31.81
	I9	32.29	32.34	32.11	32.44	32.53	30.99	32.46	32.30	31.42
	I10	32.31	32.30	31.14	31.46	31.58	31.07	31.50	31.56	31.77
19	I1	34.87	34.86	34.21	34.36	34.23	33.28	34.22	34.54	34.68
	I2	34.81	34.85	34.72	34.98	34.97	33.31	35.07	34.26	34.25
	I3	34.78	34.79	33.74	34.22	34.14	35.07	34.10	34.70	34.52
	I4	34.82	34.88	34.30	34.67	34.65	35.39	34.68	34.35	34.37
	I5	34.83	34.89	28.34	29.00	29.04	29.68	29.15	34.45	34.00
	I6	34.83	34.83	25.47	26.75	26.54	27.54	25.98	34.43	34.00
	I7	34.86	34.87	34.31	34.64	34.73	35.32	34.56	34.63	34.63
	I8	34.80	34.87	34.85	35.20	35.23	35.98	35.27	34.77	33.91
	I9	34.84	34.87	34.52	34.96	35.02	33.32	35.06	34.64	34.28
	I10	34.85	34.81	33.58	33.92	34.02	33.32	34.02	34.15	34.16
25	I1	41.66	41.77	40.65	41.07	40.64	39.56	40.96	41.69	40.85
	I2	41.73	41.75	41.83	42.19	41.87	42.92	42.13	41.16	40.92
	I3	41.76	41.72	40.03	40.61	40.25	41.68	40.42	41.54	41.47
	I4	41.80	41.81	40.99	41.56	41.22	42.46	41.69	41.75	41.29
	I5	41.72	41.78	33.14	33.84	33.72	34.75	33.99	41.73	41.25
	I6	41.72	41.70	29.27	30.47	29.62	32.05	29.29	41.20	41.53
	I7	41.67	41.73	41.17	41.59	41.49	39.55	41.55	41.38	41.62
	I8	41.67	41.78	41.89	42.34	42.11	39.73	42.35	41.00	41.30
	I9	41.65	41.79	41.52	41.89	41.99	39.56	42.13	41.60	41.30
	I10	41.79	41.70	40.22	40.82	40.50	39.77	40.77	41.16	40.88

Table 3 shows the SSIM results for all images. From this table, we can see that, at levels 6 and 17, the MPAMFO achieved the highest SSIM values in 90% of images, while the HHO is ranked second, followed by MPA and SSO, respectively. Whereas, the CS and GWO performed equally. At levels 8, the MPA obtained the best SSIM in 6 images whereas, the MPAMFO came in the second rank; however, the performance of both are similar to some extend. The HHO is ranked third. The PSO, MFO, and SSO came in the forth, fifth, and sixth ranks followed by the CS and GWO, respectively. At levels 15, the highest SSIM values are obtained by the MPAMFO in 80% of the images. The MPA and HHO performed equally, followed by GWO, CS, SSO, PSO, respectively. At levels 19, the MPAMFO is also ranked first and recorded the best SSIM values in 70% of the images. The HHO and MPA performed equally. Wheres, GWO is ranked fourth, followed by CS and SSO. At levels 25, the MPAMFO could also reach the highest SSIM values in 90% of the images, whereas, the second-best is the HHO algorithm followed by PSO, CS, and GWO. The MPA and SSO performed equally. Whereas, the GOA algorithm showed bad performance in all thresholds levels.

Table 4 records the fitness function values for all algorithms. In this measure, the MPAMFO achieved the best values in 5 images at level 6, followed by the GOA, MPA, and GWO, respectively. At levels 8, 17, and 19, the GOA achieved the highest values in 5, 5, and 4 images, respectively, followed by the MPAMFO. Whereas, the rest of the algorithms are ordered in the following sequence: MPA, GWO, CS, SSO, PSO, and MFO. At level 15, the MPAMFO reported the highest fitness values in 40% of the images followed by MPA and GWO, respectively. At level 25, The MPAMFO and MPA performed equally and obtained the best fitness values in 30% of the images for each one. Whereas, the SSO and GOA achieved the best fitness values in 20% of the images.

However, the GOA outperformed the proposed method in some images, and other measures showed the bad performance of the GOA. Therefore, the proposed method is considered the best method among the compared algorithms in image segmentation.

In general, the MPAMFO obtained the best PSNR values in 42% of the experiment, followed by the HHO with 32%. In terms of SSIM measure, the MPAMFO obtained the best values in 78% of the experiment, whereas, the MPA is ranked second with 15%. In the fitness values, the GOA showed the highest values in 35% of the experiment, followed by the MPAMFO with 32%. However, the performance of the GOA is the worst one in the other measures; it increases the fitness value without saving the qualities of the images.

**FIGURE 6.**
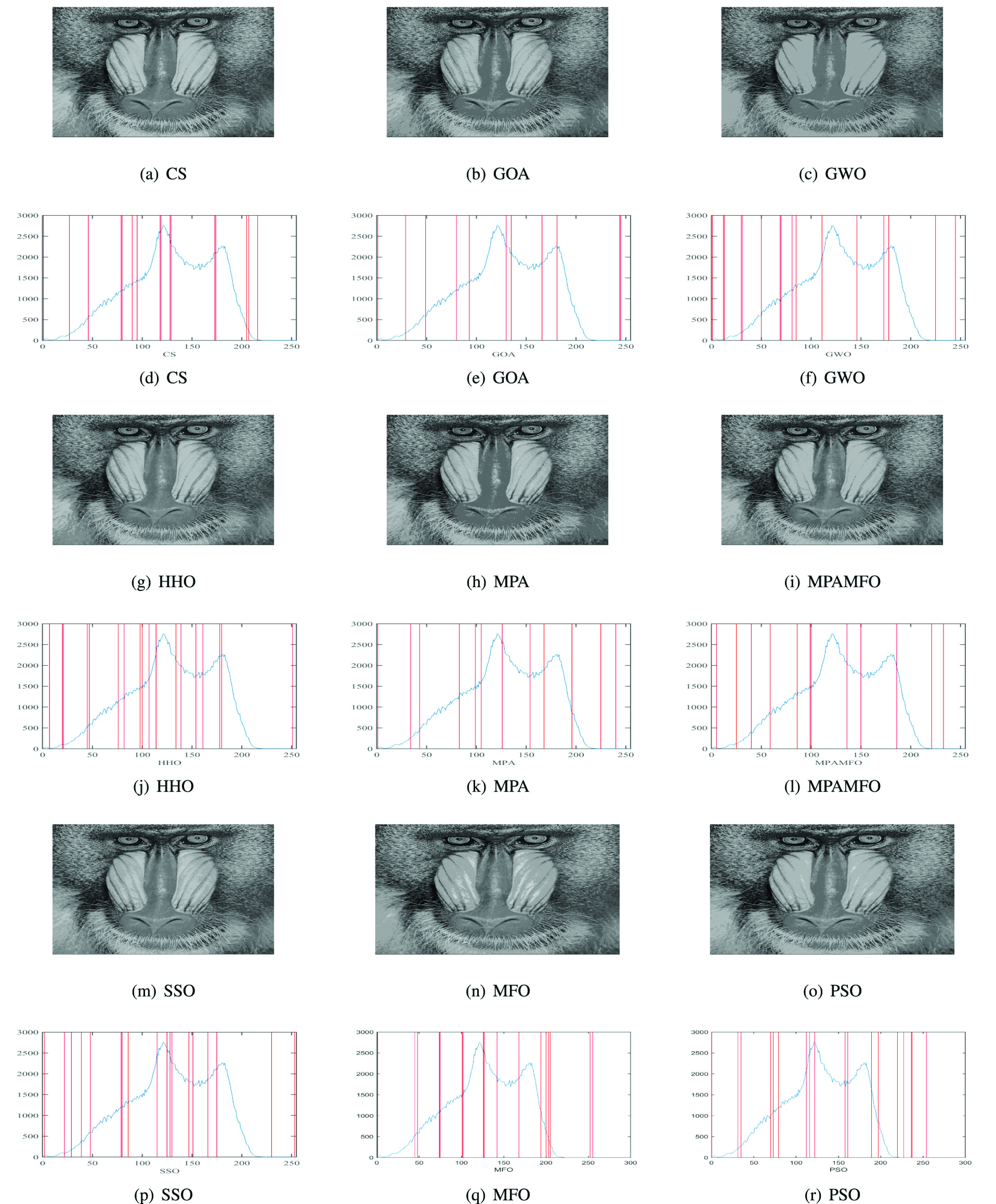
Threshold values obtained by each algorithm over the histogram of image I1.

Figure 6 depicts the threshold values obtained by each algorithm to segmented images at threshold level 19.

From the above discussion in Tables 2–4, it can be seen that the developed MPAMFO has a high ability to obtain the suitable threshold values that can be used to segment the images. However, other MH techniques used in this study fail to provide the optimal threshold values. The main reason is that most of them can stagnation at the local optimal point since they have high exploration ability with weak exploitation ability. Also, by analyzing the behavior of HHO, we see that it avoids this problem so it can provide results better than other MH algorithm since its exploitation is better than its exploration ability. Meanwhile, the proposed MPAMFO can balance between two these phases.

#### 1) Robustness of the Developed MPAMFO

To validate the robustness of MPAMFO, a set of experiments are performed using the same previous ten images under variants of three values of Gaussian noise (i.e., 0.03, 0.05, and 0.1); and at five images (I1, I3, I7, I8, and I9).

Table 5 illustrates the average of SSIM, and PSNR values for the traditional MPA and proposed MPAMFO at threshold levels 6, 16, and 19. One can be seen from these results that the proposed MPAMFO provides better results than traditional MPA in most of the tested cases, especially with increasing the level of noise. In addition, it can be observed that the performance of the two algorithms is decreased by increasing the noise level.

**TABLE 5:** Results of Study the Influence of Noise on the Quality of MPAMFO

		0.03				0.05				0.1			
Level (K)	Img	PSNR		SSIM		PSNR		SSIM		PSNR		SSIM	
		MPA	MPAMFO	MPA	MPAMFO	MPA	MPAMFO	MPA	MPAMFO	MPA	MPAMFO	MPA	MPAMFO
6	I1	13.42	14.11	0.480	0.528	13.76	14.61	0.490	0.544	13.90	14.64	0.496	0.561
	I3	9.32	10.19	0.295	0.297	9.47	10.20	0.362	0.350	9.72	10.28	0.414	0.401
	I7	11.26	12.19	0.311	0.327	11.42	12.21	0.349	0.345	11.61	12.41	0.364	0.354
	I8	11.76	14.30	0.461	0.465	13.30	14.43	0.489	0.505	13.45	14.70	0.520	0.559
	I9	11.00	11.82	0.411	0.398	11.03	11.85	0.457	0.452	11.07	11.90	0.468	0.458
15	I1	20.81	21.44	0.819	0.806	20.85	21.80	0.821	0.820	21.88	22.04	0.829	0.821
	I3	16.18	16.28	0.669	0.650	16.37	16.74	0.671	0.720	16.54	16.97	0.741	0.793
	I7	18.91	19.69	0.719	0.724	19.13	19.39	0.762	0.752	20.13	19.98	0.775	0.769
	I8	20.90	20.76	0.785	0.801	21.13	21.58	0.807	0.818	21.43	21.81	0.833	0.842
	I9	17.48	19.43	0.663	0.642	17.70	21.16	0.746	0.778	18.57	20.68	0.848	0.876
19	I1	19.24	23.84	0.832	0.872	23.60	23.85	0.853	0.888	23.80	24.45	0.864	0.894
	I3	18.18	21.70	0.721	0.748	19.23	22.35	0.766	0.847	20.29	22.75	0.827	0.874
	I7	21.70	22.70	0.807	0.814	21.77	23.55	0.818	0.817	22.90	23.66	0.826	0.829
	I8	20.03	23.69	0.827	0.823	22.91	23.72	0.851	0.859	23.96	23.53	0.869	0.879
	I9	18.06	22.57	0.734	0.733	20.10	23.03	0.809	0.828	20.70	23.28	0.832	0.873

### D. Second Experiment: Real-World Application of COVID-19 Ct Images

**FIGURE 7.**
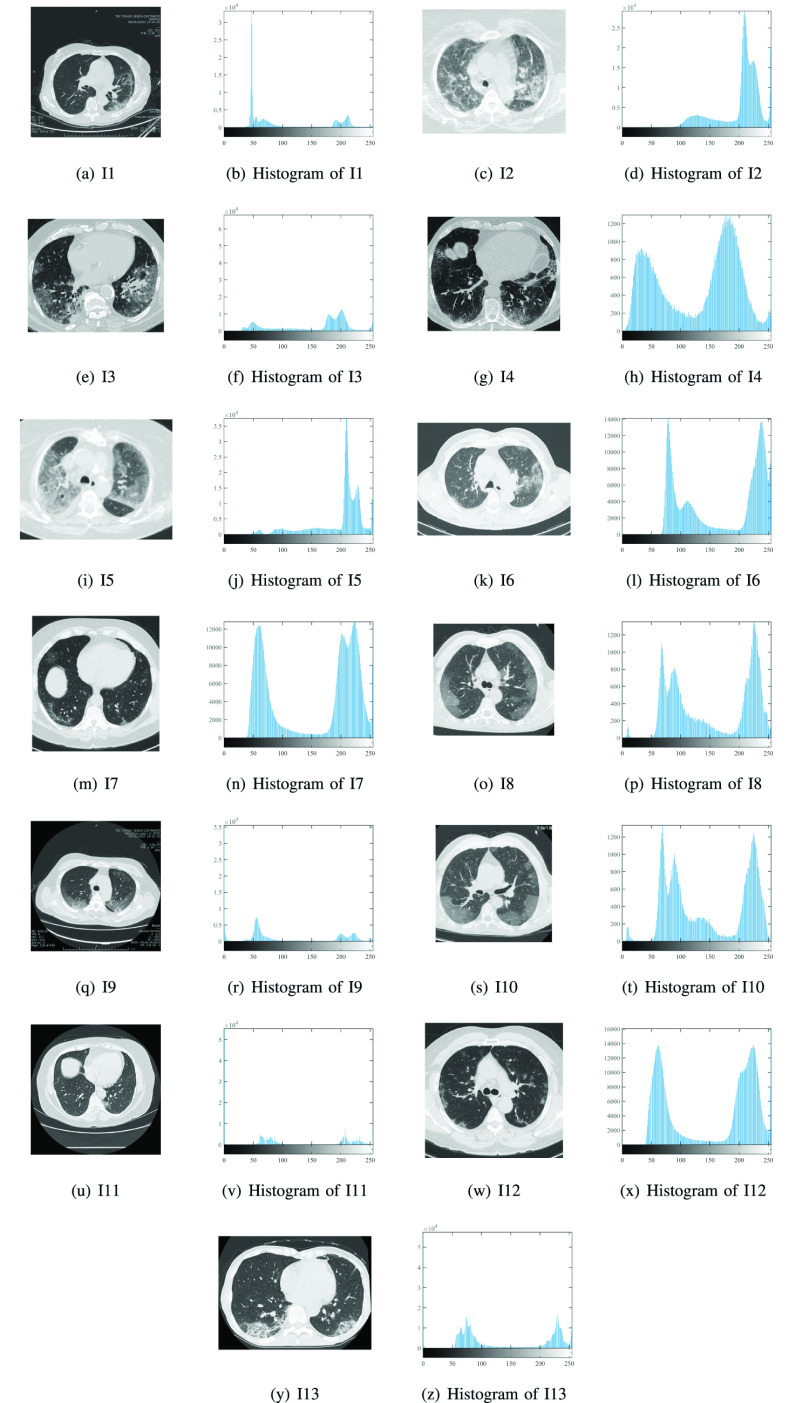
Histograms and original COVID-19 images.

To assess the quality of the segmentation method for COVID-19 CT images, a set of thirteen images is used from [53] as in Figure 7. These images are collected from different datasets such as CheX aka CheXpert [94], OpenI [95], Google [96], PC aka PadChest [97], NIH aka Chest X-ray14 [98], and MIMIC-CXR [99]. The images are resized to $224\times 224$ pixels [53]. Each of which is segmented using five thresholds’s levels (i.e. 6, 8, 15, 17, and 19). The results are recorded in Tables 6–8 and 8–10.

**TABLE 6:** Results of the PSNR Measure for All Algorithms for the Second Experiment

Level (K)	Image	MPA	MPAMFO	HHO	CS	GWO	GOA	SSO	PSO	MFO
6	Cov1	15.07	15.13	15.97	15.49	15.37	15.08	16.85	15.59	14.28
	Cov2	11.86	19.63	17.36	12.61	12.86	11.38	18.80	14.79	14.39
	Cov3	11.98	17.06	14.51	12.73	12.65	12.78	16.56	13.04	12.49
	Cov4	12.80	17.81	15.37	13.27	13.07	12.88	16.93	14.39	14.25
	Cov5	11.07	18.07	16.23	11.44	11.83	11.64	17.64	14.55	14.44
	Cov6	12.58	18.55	13.93	13.05	12.09	12.97	16.77	12.89	13.62
	Cov7	15.48	16.24	15.49	15.97	15.49	15.78	15.76	13.83	13.71
	Cov8	10.28	13.83	10.32	10.72	10.44	9.58	11.39	13.41	13.18
	Cov9	15.65	17.50	15.56	16.27	15.65	15.50	15.99	14.95	14.77
	Cov10	10.25	13.35	10.17	10.77	10.90	9.67	11.20	13.94	13.37
	Cov11	15.25	16.51	15.36	15.54	15.44	15.78	15.33	14.74	14.47
	Cov12	15.18	16.55	15.20	15.72	14.53	15.47	15.42	13.60	13.57
	Cov13	15.55	15.97	15.64	15.87	15.39	15.08	15.62	13.20	13.36
8	Cov1	16.84	22.73	20.11	17.55	18.14	17.40	19.95	19.18	18.87
	Cov2	17.09	22.41	20.52	18.57	17.65	17.01	19.64	20.75	19.57
	Cov3	16.46	20.37	17.32	17.29	16.32	16.00	18.30	18.49	19.06
	Cov4	16.12	21.08	17.69	16.56	16.60	15.69	19.08	19.50	20.71
	Cov5	16.93	21.85	18.96	17.68	17.06	16.23	19.76	18.19	20.15
	Cov6	17.26	20.16	17.25	16.87	14.22	15.22	17.72	18.08	20.11
	Cov7	17.28	17.47	17.49	18.09	16.14	16.76	17.86	17.25	16.48
	Cov8	14.79	16.20	13.80	14.34	14.07	13.50	15.41	13.93	13.71
	Cov9	16.35	18.49	16.67	17.10	17.25	17.01	17.09	16.71	15.85
	Cov10	13.58	17.09	14.59	14.99	13.97	12.76	14.84	16.78	14.49
	Cov11	15.18	18.14	15.22	15.49	15.80	15.35	15.24	21.88	23.46
	Cov12	17.25	17.46	17.12	17.62	15.81	16.87	17.69	17.05	15.27
	Cov13	17.07	17.63	16.33	17.60	18.50	15.85	18.08	16.04	16.45
15	Cov1	24.06	24.24	24.02	24.39	24.10	23.29	23.89	22.54	21.80
	Cov2	22.72	24.49	26.47	24.86	22.52	21.47	24.99	22.00	23.38
	Cov3	20.58	23.77	21.89	21.16	20.86	18.87	23.28	21.21	22.75
	Cov4	20.54	23.68	21.87	21.36	21.49	18.89	22.16	22.95	22.72
	Cov5	21.70	24.27	24.89	23.63	21.68	20.15	23.31	22.81	23.36
	Cov6	18.81	23.76	18.91	20.19	17.34	16.24	21.93	21.92	22.00
	Cov7	18.19	21.20	18.59	19.72	17.87	16.61	18.73	18.17	17.25
	Cov8	19.00	21.44	19.32	20.74	19.77	17.88	20.39	16.16	17.92
	Cov9	22.05	22.40	22.53	20.84	21.92	22.03	22.36	20.13	20.04
	Cov10	19.81	22.39	19.29	20.78	19.40	18.98	21.01	17.68	18.42
	Cov11	22.19	21.36	21.67	21.60	19.22	20.89	21.40	20.06	20.82
	Cov12	18.09	20.20	18.72	19.49	17.54	16.68	18.82	21.53	22.54
	Cov13	20.00	19.90	20.61	20.41	19.50	19.83	21.93	18.44	17.48
17	Cov1	24.62	26.88	25.47	24.99	24.33	23.92	24.83	23.00	22.89
	Cov2	24.07	26.48	26.64	26.00	22.96	22.01	26.12	23.30	23.85
	Cov3	21.25	24.38	23.66	22.83	21.65	19.56	24.06	22.13	23.86
	Cov4	22.15	25.32	23.40	22.44	22.50	20.37	22.33	23.08	23.18
	Cov5	22.75	25.11	25.76	25.00	23.07	22.21	26.22	23.97	24.28
	Cov6	19.60	24.34	21.96	18.98	18.01	18.43	24.09	22.73	22.49
	Cov7	19.36	21.47	20.06	21.30	19.28	17.30	20.75	19.45	19.63
	Cov8	21.19	23.05	19.75	22.26	21.33	19.72	21.58	17.66	18.77
	Cov9	23.80	23.83	22.75	22.56	23.25	22.24	23.36	21.14	22.45
	Cov10	21.04	22.56	20.65	22.68	20.87	19.94	22.73	18.42	18.85
	Cov11	22.00	22.55	22.56	22.18	21.07	20.77	22.18	19.25	19.78
	Cov12	19.53	22.79	19.79	20.19	19.48	17.02	20.10	20.78	22.77
	Cov13	20.62	22.60	20.43	22.13	20.10	20.97	22.03	20.41	20.04
19	Cov1	25.50	27.49	26.58	26.80	25.15	24.45	26.10	26.06	26.18
	Cov2	24.75	28.42	27.29	26.39	24.12	23.50	26.37	26.47	26.78
	Cov3	22.04	26.68	24.75	23.43	23.22	20.28	25.16	26.11	26.30
	Cov4	23.60	26.08	24.95	23.64	24.05	21.48	25.06	25.86	25.31
	Cov5	23.95	26.39	26.41	26.26	23.66	22.92	26.36	25.24	25.82
	Cov6	20.51	26.35	22.26	19.89	19.30	18.72	25.59	24.15	26.46
	Cov7	20.77	23.33	20.97	19.68	20.00	18.23	22.05	20.67	22.56
	Cov8	22.59	24.20	22.52	24.07	22.28	21.18	23.18	20.14	20.85
	Cov9	23.82	25.94	24.17	25.02	24.36	23.25	23.54	22.94	22.13
	Cov10	22.42	24.70	21.33	24.00	21.99	21.85	23.52	20.30	20.41
	Cov11	23.05	23.82	23.30	22.45	22.89	21.59	22.79	20.78	20.24
	Cov12	20.35	23.95	20.29	20.06	21.74	17.65	22.20	20.82	23.06
	Cov13	21.73	23.28	22.31	22.79	21.48	21.96	22.75	22.60	20.77

**TABLE 7:** Results of the SSIM Measure for All Algorithms for the Second Experiment

Level (K)	Image	MPA	MPAMFO	HHO	CS	GWO	GOA	SSO	PSO	MFO
6	Cov1	0.399	0.496	0.473	0.447	0.447	0.481	0.415	0.483	0.452
	Cov2	0.653	0.757	0.745	0.669	0.670	0.629	0.647	0.716	0.726
	Cov3	0.510	0.665	0.618	0.551	0.508	0.529	0.509	0.502	0.459
	Cov4	0.243	0.585	0.453	0.259	0.249	0.243	0.243	0.348	0.361
	Cov5	0.661	0.764	0.732	0.663	0.686	0.652	0.657	0.746	0.749
	Cov6	0.529	0.551	0.499	0.536	0.485	0.545	0.530	0.477	0.480
	Cov7	0.443	0.468	0.440	0.453	0.454	0.441	0.455	0.364	0.359
	Cov8	0.405	0.518	0.409	0.433	0.414	0.339	0.444	0.426	0.506
	Cov9	0.558	0.579	0.571	0.571	0.575	0.567	0.555	0.480	0.474
	Cov10	0.383	0.525	0.374	0.412	0.441	0.333	0.437	0.505	0.507
	Cov11	0.527	0.556	0.530	0.528	0.528	0.537	0.523	0.543	0.519
	Cov12	0.428	0.484	0.431	0.438	0.421	0.429	0.442	0.356	0.352
	Cov13	0.464	0.527	0.462	0.472	0.434	0.461	0.483	0.454	0.497
8	Cov1	0.542	0.710	0.713	0.571	0.811	0.613	0.500	0.677	0.692
	Cov2	0.752	0.760	0.785	0.757	0.754	0.737	0.753	0.726	0.754
	Cov3	0.672	0.696	0.687	0.686	0.641	0.600	0.658	0.647	0.680
	Cov4	0.503	0.694	0.586	0.540	0.538	0.500	0.510	0.634	0.675
	Cov5	0.755	0.800	0.776	0.768	0.767	0.737	0.760	0.783	0.771
	Cov6	0.602	0.598	0.570	0.603	0.477	0.500	0.598	0.559	0.568
	Cov7	0.533	0.517	0.546	0.542	0.467	0.488	0.544	0.522	0.521
	Cov8	0.551	0.594	0.503	0.520	0.521	0.479	0.568	0.514	0.532
	Cov9	0.510	0.587	0.525	0.537	0.558	0.532	0.545	0.570	0.520
	Cov10	0.505	0.606	0.557	0.574	0.537	0.448	0.550	0.570	0.571
	Cov11	0.520	0.626	0.522	0.531	0.546	0.524	0.523	0.608	0.601
	Cov12	0.533	0.511	0.518	0.535	0.446	0.499	0.536	0.519	0.501
	Cov13	0.582	0.608	0.546	0.596	0.654	0.536	0.615	0.617	0.612
15	Cov1	0.863	0.846	0.855	0.856	0.866	0.836	0.865	0.817	0.805
	Cov2	0.814	0.832	0.842	0.818	0.818	0.779	0.807	0.797	0.786
	Cov3	0.692	0.782	0.737	0.720	0.702	0.643	0.709	0.696	0.722
	Cov4	0.763	0.816	0.806	0.785	0.814	0.691	0.773	0.748	0.777
	Cov5	0.817	0.819	0.814	0.828	0.829	0.777	0.820	0.803	0.795
	Cov6	0.625	0.720	0.646	0.675	0.574	0.523	0.587	0.740	0.711
	Cov7	0.554	0.679	0.580	0.621	0.528	0.483	0.572	0.646	0.630
	Cov8	0.674	0.737	0.679	0.712	0.714	0.622	0.707	0.721	0.737
	Cov9	0.739	0.747	0.751	0.709	0.756	0.731	0.741	0.765	0.770
	Cov10	0.724	0.761	0.707	0.737	0.735	0.697	0.751	0.720	0.727
	Cov11	0.771	0.749	0.752	0.762	0.698	0.741	0.750	0.726	0.705
	Cov12	0.553	0.629	0.586	0.618	0.514	0.485	0.575	0.627	0.610
	Cov13	0.707	0.685	0.720	0.715	0.707	0.732	0.742	0.726	0.727
17	Cov1	0.863	0.856	0.871	0.870	0.867	0.855	0.862	0.833	0.840
	Cov2	0.811	0.842	0.837	0.829	0.829	0.795	0.818	0.818	0.806
	Cov3	0.713	0.785	0.784	0.758	0.731	0.660	0.713	0.728	0.746
	Cov4	0.833	0.828	0.860	0.827	0.851	0.765	0.831	0.750	0.785
	Cov5	0.831	0.851	0.844	0.838	0.835	0.813	0.840	0.813	0.811
	Cov6	0.646	0.775	0.723	0.663	0.605	0.608	0.628	0.751	0.615
	Cov7	0.597	0.676	0.626	0.682	0.585	0.516	0.638	0.650	0.636
	Cov8	0.736	0.778	0.696	0.763	0.748	0.699	0.747	0.737	0.748
	Cov9	0.793	0.779	0.759	0.754	0.800	0.743	0.758	0.785	0.781
	Cov10	0.764	0.778	0.749	0.775	0.758	0.718	0.781	0.747	0.730
	Cov11	0.767	0.779	0.785	0.782	0.746	0.733	0.767	0.742	0.727
	Cov12	0.613	0.715	0.622	0.654	0.597	0.509	0.627	0.630	0.618
	Cov13	0.738	0.761	0.731	0.754	0.737	0.749	0.743	0.730	0.739
19	Cov1	0.870	0.880	0.889	0.894	0.885	0.859	0.872	0.849	0.858
	Cov2	0.820	0.837	0.845	0.844	0.835	0.800	0.830	0.805	0.813
	Cov3	0.734	0.820	0.797	0.770	0.761	0.683	0.740	0.808	0.758
	Cov4	0.872	0.894	0.897	0.857	0.886	0.802	0.856	0.856	0.833
	Cov5	0.835	0.858	0.843	0.857	0.840	0.817	0.838	0.839	0.833
	Cov6	0.674	0.803	0.728	0.692	0.639	0.625	0.648	0.770	0.751
	Cov7	0.644	0.743	0.659	0.629	0.610	0.558	0.691	0.708	0.745
	Cov8	0.774	0.806	0.773	0.796	0.772	0.743	0.777	0.823	0.781
	Cov9	0.802	0.832	0.803	0.809	0.828	0.768	0.768	0.812	0.804
	Cov10	0.779	0.817	0.764	0.806	0.771	0.768	0.800	0.786	0.762
	Cov11	0.790	0.814	0.793	0.785	0.789	0.757	0.775	0.758	0.747
	Cov12	0.646	0.753	0.644	0.661	0.673	0.548	0.700	0.722	0.734
	Cov13	0.766	0.782	0.772	0.772	0.759	0.767	0.777	0.737	0.759

**TABLE 8:** Results of the Fitness Function Value for All Algorithms for the Second Experiment

Level (K)	Image	MPA	MPAMFO	HHO	CS	GWO	GOA	SSO	PSO	MFO
6	Cov1	15.740	15.750	15.630	15.720	15.730	15.720	15.430	14.991	15.663
	Cov2	16.450	16.460	16.220	16.460	16.460	16.500	15.940	16.276	15.825
	Cov3	16.760	16.780	16.570	16.760	16.760	16.770	16.350	16.777	16.156
	Cov4	18.020	18.020	17.840	18.060	18.080	18.090	17.560	17.483	17.964
	Cov5	16.900	16.910	16.650	16.880	16.870	16.900	16.420	16.507	16.522
	Cov6	16.450	16.460	16.240	16.400	16.390	16.440	15.960	16.272	15.636
	Cov7	16.853	16.613	16.856	16.816	16.814	16.850	16.784	16.520	15.963
	Cov8	16.908	16.736	16.912	16.893	16.896	16.918	16.863	16.443	16.011
	Cov9	16.272	16.325	16.274	16.222	16.232	16.249	16.238	16.288	15.877
	Cov10	16.937	16.826	16.938	16.955	16.956	16.984	16.899	16.727	16.771
	Cov11	15.230	14.919	15.232	15.117	15.124	15.013	15.182	14.742	14.256
	Cov12	16.765	16.604	16.765	16.817	16.780	16.860	16.695	15.625	15.989
	Cov13	16.359	16.202	16.362	16.316	16.310	16.325	16.316	15.736	15.357
8	Cov1	19.190	19.210	18.870	19.080	19.100	19.170	18.640	18.740	18.569
	Cov2	19.860	19.880	19.340	19.780	19.850	19.760	18.880	19.074	19.411
	Cov3	20.000	20.020	19.760	19.930	19.960	20.030	19.220	19.987	19.593
	Cov4	21.565	21.550	21.290	21.470	21.520	21.560	20.830	21.497	20.759
	Cov5	20.230	20.240	19.850	20.160	20.170	20.280	19.440	19.536	19.564
	Cov6	19.670	19.700	19.430	19.610	19.580	19.670	18.930	18.966	18.797
	Cov7	20.369	20.251	20.372	20.288	20.299	20.362	20.288	19.764	20.062
	Cov8	20.318	20.211	20.317	20.246	20.273	20.313	20.167	19.812	19.675
	Cov9	19.854	19.585	19.846	19.732	19.792	19.839	19.680	19.298	18.751
	Cov10	20.326	20.995	20.345	20.277	20.232	20.351	20.222	20.916	20.844
	Cov11	18.599	18.172	18.592	18.452	18.477	18.464	18.359	18.110	17.300
	Cov12	20.353	20.117	20.367	20.304	20.297	20.336	20.284	19.578	19.309
	Cov13	19.286	19.905	19.713	19.632	19.602	19.732	19.580	19.170	19.515
15	Cov1	28.560	28.590	27.770	28.220	28.340	28.580	27.200	28.004	28.169
	Cov2	28.390	28.490	26.590	27.780	27.620	28.260	25.820	28.468	27.929
	Cov3	29.700	29.730	28.950	29.270	29.330	29.890	28.140	28.776	28.859
	Cov4	30.800	30.800	30.070	30.480	30.470	30.930	29.430	30.403	29.859
	Cov5	28.990	28.970	27.880	28.520	28.850	29.150	27.000	28.310	28.442
	Cov6	28.400	28.520	27.360	27.780	27.600	28.320	25.240	28.238	27.975
	Cov7	29.490	28.405	29.535	29.040	29.329	29.458	28.863	27.581	28.230
	Cov8	29.174	28.253	29.318	28.742	28.676	29.281	28.755	27.266	27.930
	Cov9	28.625	27.541	28.716	28.079	28.002	28.706	28.027	26.649	26.682
	Cov10	29.348	28.462	29.385	28.953	29.056	29.481	28.969	27.927	28.041
	Cov11	26.824	27.038	27.014	26.201	25.894	26.795	26.002	26.652	26.560
	Cov12	29.532	29.564	29.557	29.086	29.352	29.507	29.005	29.368	29.463
	Cov13	28.440	27.234	28.577	28.058	27.256	27.869	27.816	26.901	26.535
17	Cov1	31.260	31.340	30.190	30.740	30.760	31.220	29.650	30.789	31.099
	Cov2	30.970	30.940	29.210	30.110	29.400	30.560	27.380	30.706	30.821
	Cov3	32.340	32.350	31.460	31.870	31.940	32.490	30.530	31.416	32.021
	Cov4	33.490	33.620	32.620	33.110	33.080	33.700	32.090	33.549	33.163
	Cov5	31.580	31.630	30.300	30.910	31.250	31.610	29.200	31.338	30.751
	Cov6	30.960	30.970	28.560	30.170	29.800	30.790	27.670	30.646	30.050
	Cov7	32.142	31.127	32.196	31.491	31.601	32.314	31.378	30.485	30.977
	Cov8	31.786	30.751	31.810	31.212	31.124	31.775	31.266	30.182	30.384
	Cov9	31.022	29.705	31.189	30.479	30.198	30.883	30.526	29.510	29.694
	Cov10	32.111	32.276	32.085	31.556	31.595	32.274	31.644	31.358	32.050
	Cov11	29.161	27.434	29.214	28.161	28.111	29.388	28.266	27.286	26.467
	Cov12	32.165	32.928	32.231	31.619	31.730	32.188	31.388	32.465	32.834
	Cov13	30.470	29.705	31.055	30.247	29.987	30.323	30.202	28.718	29.003
19	Cov1	33.780	33.790	32.740	33.320	33.360	33.730	32.190	33.361	32.859
	Cov2	33.250	33.470	31.500	32.230	31.660	33.050	29.080	32.650	33.393
	Cov3	34.900	34.860	33.670	34.330	34.410	35.050	32.490	34.330	34.556
	Cov4	36.160	36.230	35.270	35.740	35.590	36.390	34.530	35.364	35.505
	Cov5	34.050	34.010	32.530	33.300	33.320	33.770	31.200	33.482	33.470
	Cov6	33.280	33.340	31.210	32.370	31.830	32.550	28.810	32.717	33.064
	Cov7	34.675	33.410	34.745	33.945	33.884	34.718	33.658	33.352	33.290
	Cov8	34.229	33.418	34.228	33.663	33.530	34.180	33.774	33.212	32.918
	Cov9	33.341	32.178	33.420	32.873	32.749	33.346	32.879	31.814	31.389
	Cov10	34.720	34.781	34.778	34.135	34.215	34.760	34.270	34.048	34.502
	Cov11	31.494	29.853	31.350	30.351	30.217	31.305	30.263	29.479	28.971
	Cov12	34.798	33.497	34.735	33.999	34.085	34.540	33.608	33.274	33.341
	Cov13	33.104	32.291	33.308	32.576	32.642	33.093	32.683	31.729	31.982

**TABLE 9:** Friedman Test Results for the First Experiment

	MPA	MPAMFO	HHO	CS	GWO	GOA	SSO	PSO	MFO
PSNR	4.43	7.93	7.57	5.40	3.75	1.55	5.07	5.25	4.05
SSIM	5.93	8.67	6.91	5.15	5.01	1.62	4.43	4.18	3.12

**TABLE 10:** Friedman Test Results for the Second Experiment

	MPA	MPAMFO	HHO	CS	GWO	GOA	SSO	PSO	MFO
PSNR	3.76	8.38	5.75	5.70	3.71	2.38	6.64	4.32	4.35
SSIM	3.95	7.89	5.62	5.97	4.86	2.31	4.95	4.82	4.64

**FIGURE 8.**
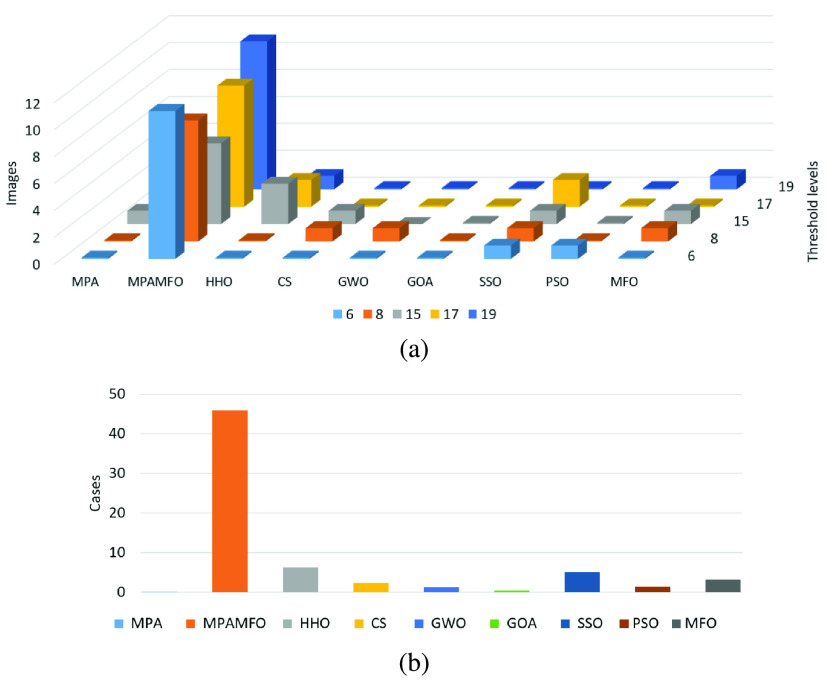
Summary of the PSNR results for the second experiment. (a) illustrates the performance of each algorithm at thresholds levels. (b) illustrates the numbers of the best cases obtained by each algorithm.

Table 6 shows the results of the PSNR measure for the images. The results indicate that the MPAMFO obtained the best PSNR values in 11 images at the threshold level 6 whereas, the SSO and PSO got the best results in only one image for each one and they are ranked second and third, respectively. The HHO and CS obtained the fourth and fifth rank. The MPAMFO outperformed all other algorithms at level 8, and it obtained the best PSNR values in 69% of the images. The MFO is ranked second, followed by PSO, SSO, HHO, CS, GWO, and MPA, respectively. At levels 15 and 19, the MFO got the second rank after the MPAMFO then the CS came third. The rest of the algorithms were ordered as follows, SSO, HHO, PSO, MPA, then GWO, while the GOA showed the worst performance in all images. At level 17, the MPAMFO produced the best results in 9 images, whereas, the HHO and SSO performed equally with two images for each one. The CS was ranked fourth. While the MFO and MPA showed the same performance in most images. The GOA showed the worst performance in all images at all threshold levels. At all levels, the MPAMFO obtained the best values in 46 out of 65 cases (13 images and five threshold levels), as shown in Figure 8.

**FIGURE 9.**
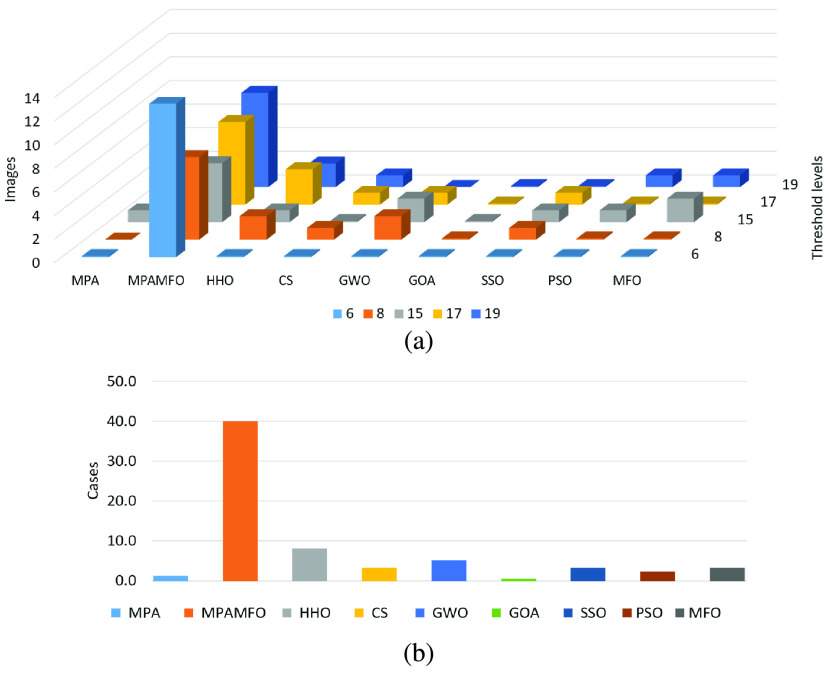
Summary of the SSIM results for the second experiment. (a) illustrates the performance of each algorithm at thresholds levels. (b) illustrates the numbers of the best cases obtained by each algorithm.

To analyze the SSIM results, Table 7 and Figure 9 report that the MPAMFO is ranked first at all thresholds levels. It recorded the best SSIM values in 13, 7, 5, 7, and 8 images at thresholds levels 6, 8, 15, 17, and 19, respectively, and achieved the best SSIM in 61% of all cases. The HHO is ranked second at levels 17 and 19. In these levels, the CS and GWO obtained the third and fourth rank, followed by SSO and PSO, respectively. At level 8, the HHO showed the best performance after the MPAMFO, followed by CS and PSO, respectively. At level 15, the GWO produced the best SSIM values in three images, whereas, the HHO showed the best results in one image. The rest of the algorithms showed similar performance except GOA.

**FIGURE 10.**
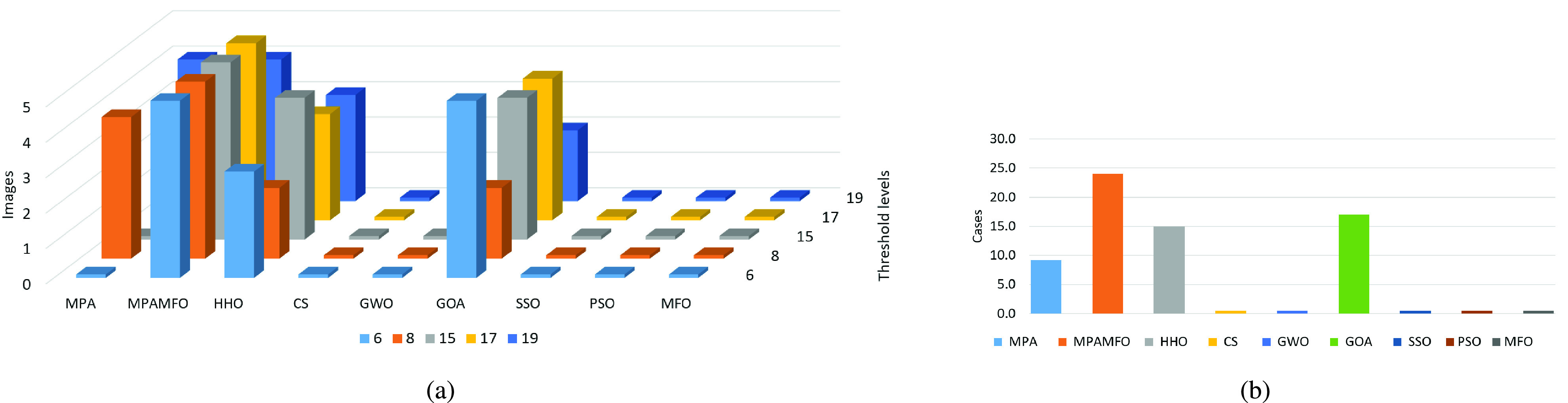
Summary of the fitness value results for the second experiment. (a) illustrates the performance of each algorithm at thresholds levels. (b) illustrates the numbers of the best cases obtained by each algorithm.

The fitness function value is also analyzed and the results are listed in Table 8 and Figure 10. These results show that the MPAMFO obtained the highest fitness values at levels 6, 15, and 17 while the GOA came second, followed by HHO, MPA, and GWO. At levels 8 and 19, the MPAMFO performed similarly as MPA; however, the average of the fitness values for the MPAMFO is lightly higher than those of the MPA. The GWO and HHO were ranked third and fourth, respectively, followed by GOA, CS, PSO, and MFO.

In general, the MPAMFO obtained the best PSNR values in 70% of the experiment, followed by the HHO with 9% of the images. In terms of SSIM measure, the MPAMFO obtained the best values in 61% of the images followed by the HHO and GWO with 12% and 8% of the images, respectively. The MPAMFO also achieved the highest values in the fitness values in 36% of all images, whereas, GOA obtained the second-best in 25% of the images followed by HHO.

Figure 12 depicts the threshold values obtained by each algorithm to segmented image I1 for COVID-19.

**FIGURE 11.**
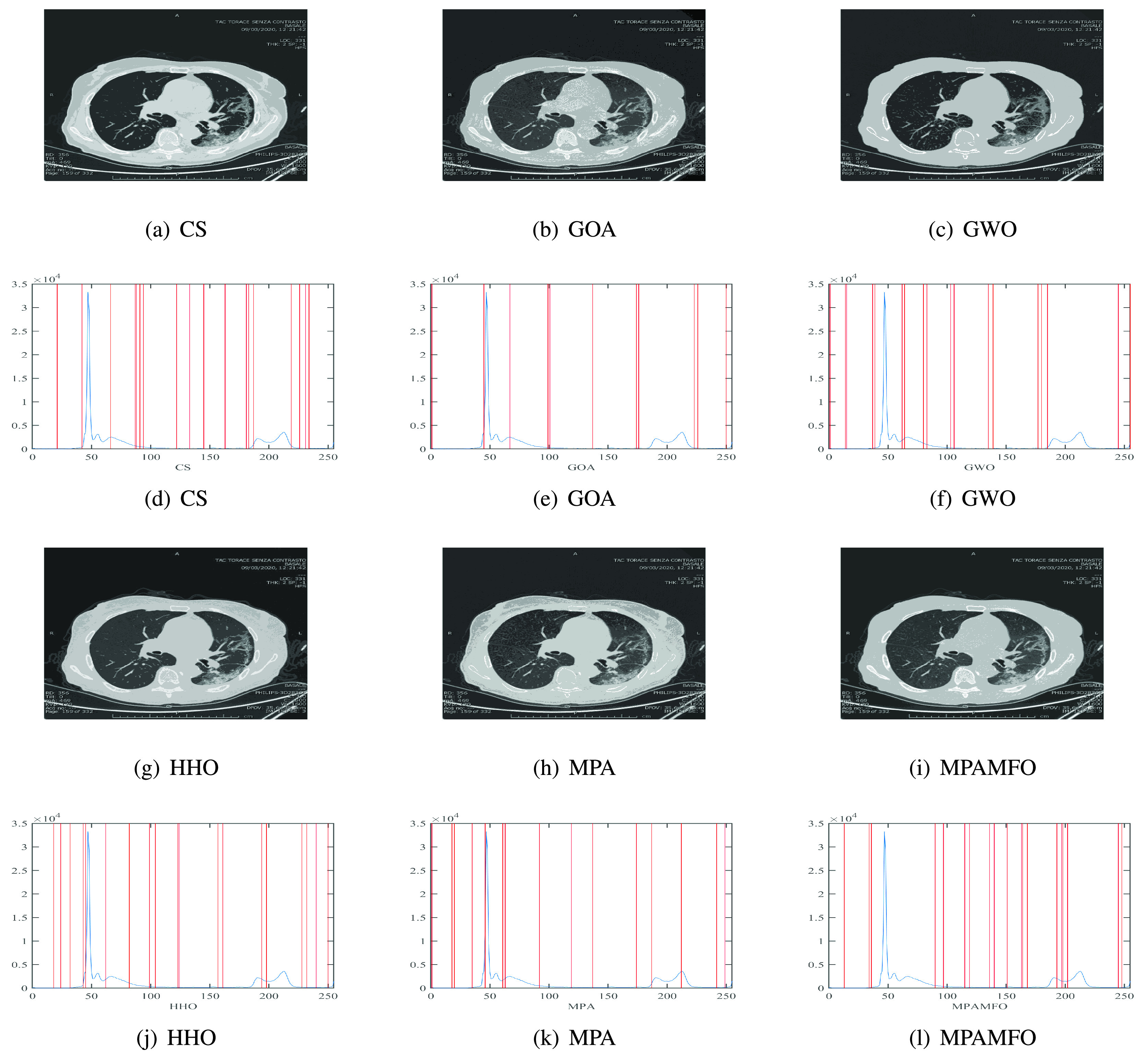
Segmented image and Threshold values obtained by each algorithm over the histogram of image I1 for CoVID-19.

**FIGURE 12.**
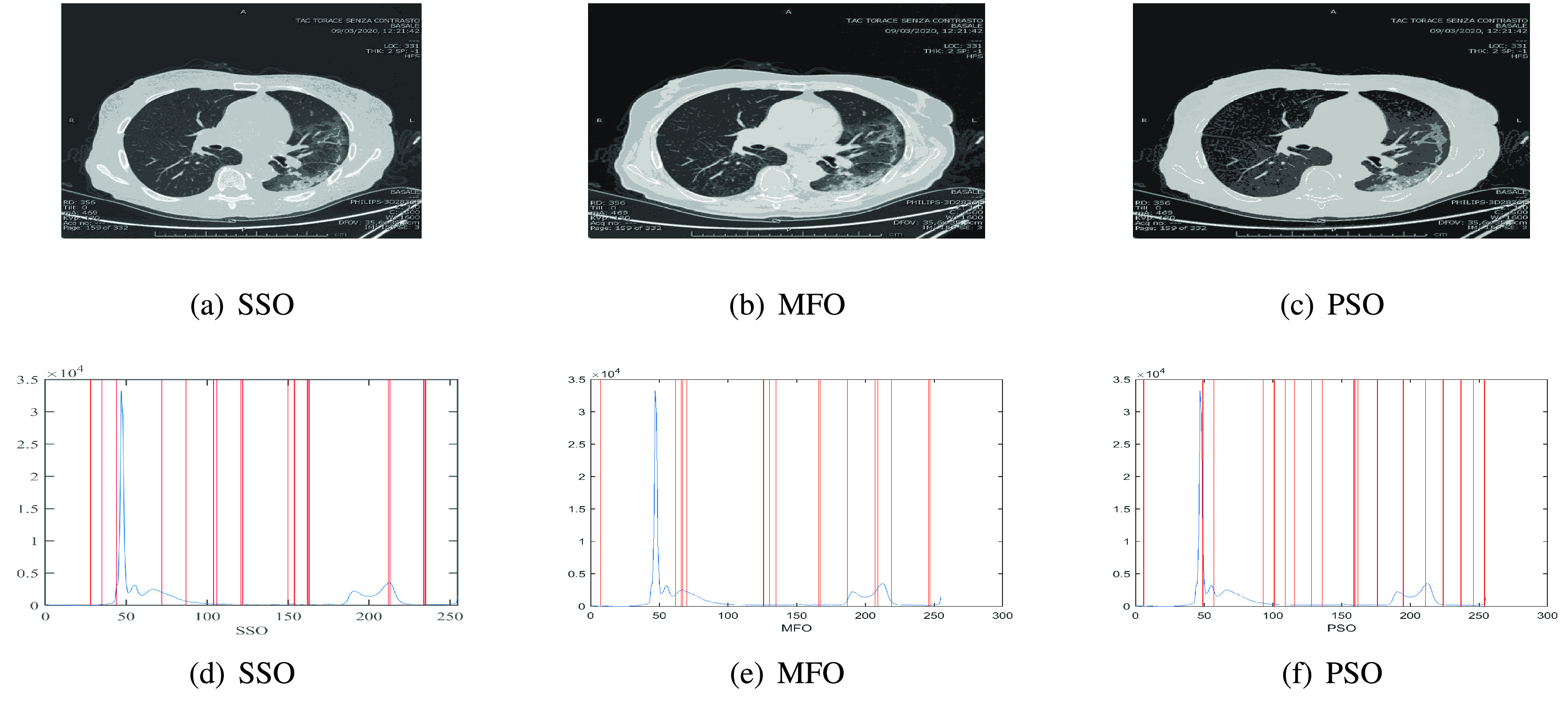
Segmented image and Threshold values obtained by each algorithm over the histogram of image I1 for CoVID-19.

### E. Statistical Results

In this section, we applied Friedman test to study the robustness of all algorithms in the experiments. The Friedman test statistically ranks the algorithms. In this rank, the highest value is the best. The results of first and second experiments are listed in Table 9 and X, respectively.

From Table 9, the MPAMFO algorithm obtained the highest mean rank among the two measures (i.e., PSNR and SSIM), followed by the HHO, CS, SSO, PSO, MPA, and MFO, respectively, in the PSNR measure; and the HHO, MPA, CS, GWO, SSO, PSO, and MFO, respectively, in the SSIM measure. For the second experiment, Table 10 shows that the MPAMFO algorithm also has the highest rank in both measures, followed by SSO and HHO. Whereas, CS, MFO, PSO, and MPA, and GWO allocate from the fourth to eighth ranks, respectively according to PSNR measure. Meanwhile, based on the SSIM value, the algorithms are ranked as in the following order, the CS, HHO, SSO, GWO, PSO, and MFO, respectively. From these two tables, it can see that GOA is the worst result according to the results of the experiments.

For further analysis, the Wilcoxon rank-sum test is used to check the statistical differences between the proposed method and the compared algorithms as in Tables 11 and 12. From Table 11, there are statistical differences between MPAMFO and MPA, GWO, GOA, and MFO based on the PSNR measure. Whereas, based on the SSIM measure, there are statistical differences between MPAMFO and GOA, SSO, PSO, and MFO. From Table 12, the MPAMFO showed statistical differences with all algorithms in both measure except the SSO for the PSNR, and HHO, CS, and PSO for the SSIM measure.

**TABLE 11:** Wilcoxon Rank Sum Test Results for the First Experiment

	MPA	HHO	CS	GWO	GOA	SSO	PSO	MFO
PSNR	0.049	0.783	0.214	0.035	0.000	0.177	0.218	0.048
SSIM	0.132	0.291	0.065	0.056	0.000	0.034	0.040	0.005

**TABLE 12:** Wilcoxon Rank Sum Test Results for the Second Experiment

	MPA	HHO	CS	GWO	GOA	SSO	PSO	MFO
PSNR	0.000	0.016	0.008	0.000	0.000	0.108	0.001	0.006
SSIM	0.027	0.153	0.127	0.047	0.000	0.037	0.075	0.049

From the above two experimental series, it can be observed the superiority of the developed MPAMFO overall the compared algorithms. However, MPAMFO has some limitations that need to be improved; for example, complexity is higher than the original MPA. Since it depends on MFO (during exploration phase) that using the sorting process during searching about the optimal threshold values, and this performed by using Quicksort algorithm. In addition, the initial population affects the quality of the final output, and for fixing this point, the chaotic maps or opposite-based learning techniques can be used.

## VII. Conclusions

This paper presents an efficient multi-level thresholding (MLT) method for image segmentation including medical image segmentation, such as COVID-19 CT images. The proposed method uses a new swarm intelligence (SI) method, called marine predators algorithm (MPA). The MPA is a novel SI method, and therefore, for our knowledge, this study presents the first application of the MPA for image segmentation. The MPA is improved using the moth-?ame optimization (MFO) algorithm. The operators of the MFO are applied to improve the exploitation ability of the MPA by working as a local search of the MPA. The proposed MPAMFO was evaluated with different images, including CT images of new coronavirus (COVID-19), and it showed good and stable performances in all tests. More so, extensive comparisons were implemented to approve the superiority of the proposed MPAMFO over several existing methods, such as GWO, SSA, CS, PSO, and the originals MFO and MPA. Evaluation outcomes showed that the MPAMFO outperforms other methods in terms of SSIM, PSNR, and fitness value.

Overall, the proposed MPAMFO assesses its high performance; therefore, in the future, it could be improved to be applied in various optimization applications, such as time series forecasting, data clustering, cloud computing, machine job scheduling, and others. Also, for COVID-19 CT image segmentation, there are several algorithms can be considered in the future work, such as improving MPAMFO as a multi-objective image segmentation method, using recent new MH technique such as Henry Gas optimization algorithm, and Slime mould algorithm.
